# Reconsidering Excellence in the Chemical Sciences: Lessons from First-Generation Chemists

**DOI:** 10.1021/acscentsci.6c00822

**Published:** 2026-09-23

**Authors:** Robert A. Ives, David N. Azulay, Adam Cook, Mamta Dagar, Douglas J. Fansher, Roshan Keshari, Anupam Anand Ojha, Shivani Patel, Christian Sandoval-Pauker, Nitesh Sanghai, Brittany K. Trinh, Vishal Yadav, Zhiling Zheng

**Affiliations:** a Department of Biochemistry, 2152 University of Cambridge, Cambridge CB2 1GA, U.K.; b School of Chemistry, Faculty of Exact Sciences, The Center for Nanoscience and Nanotechnology, The Center for Physics and Chemistry of Living Systems, Tel Aviv University, Ramat Aviv, Tel Aviv 6139001, Israel; c Department of Chemistry, 6429 Stanford University, Stanford, California 94305, United States; d Department of Chemistry, The University of Texas at Austin, Austin, Texas 78712-1224, United States; e1 Department of Chemistry, Université de Montréal, 1375 Thérèse-Lavoie-Roux ave, Montréal, QC H2 V 0B3, Canada; e2 PROTEO, The Québec Network for Protein Function, Engineering and Applications, Montréal, QC G1 V 0A6, Canada; e3 CGCC, Center in Green Chemistry and Catalysis, Montreal, QC H2 V 0B3, Canada; f Indian Institute of Technology (IIT) Bombay, Powai, Mumbai 400 076, Maharashtra India; g Center for Computational Biology and Center for Computational Mathematics, 525571 Flatiron Institute, 162 Fifth Avenue, New York, New York 10010, United States; h Department of Chemistry, 5755 Yale University, New Haven, Connecticut 06520-8107, United States; i Smalley-Curl Institute, 3990 Rice University, Houston, Texas 77005, United States; j College of Pharmacy, Rady Faculty of Health Science, 8664 University of Manitoba, Winnipeg, MB R3E 0T5, Canada; k Department of Chemistry, 5228 University of Wisconsin−Madison, Madison, Wisconsin 53706, United States; l Department of Chemistry, The Pennsylvania State University, University Park, Pennsylvania 16802, United States; m Department of Chemistry, Washington University, St. Louis, Missouri 63130, United States

Excellence in academia has long been quantified through metrics such as publication output, research funding, institutional and journal prestige, and citation impact.^1–5^ While these indicators capture important aspects of scientific productivity, they often overlook the diverse pathways through which scientists contribute to knowledge, innovation, and the broader scientific community. The experiences of first-generation chemists—defined in this editorial as individuals who are the first in their immediate families to attend university and pursue higher education in the chemical sciences—offer an opportunity to reflect on how excellence is expressed in practice. While the experiences of first-generation chemists are diverse, those who are also the first in their family to attend university (irrespective of subject field) may encounter additional educational, socioeconomic, and cultural barriers that further impact their academic journey. The experiences discussed highlight how excellence can be reconsidered in environments that were not originally designed to accommodate such trajectories. This editorial has been developed by members of the American Chemical Society’s CAS 2025 Future Leaders Top 100 cohort^6^—an initiative aimed to connect and empower emerging early career scientists from around the world to take on leadership roles and contribute to the future of the chemical sciences. Themes addressed in this editorial have been identified through structured writing reflections, group discussions, and collaborative revisions by all authors. To build this editorial, we asked ourselves four questions that are the pillars of what is being discussed herein: 1) What is meant by excellence in the chemical sciences, and whether it is necessary to reassess its meaning? 2) What are the pathways and barriers for first-generation chemists? 3) What are the forms of excellence demonstrated by first-generation chemists? 4) How might insights from first-generation chemists inform structural changes in the chemical sciences?

Drawing on our collective reflections and shared experiences, this editorial explores these central themes that shape the experiences of first-generation chemists. By examining these interconnected issues, we discuss how current evaluation systems may overlook important dimensions of scientific contribution and consider opportunities to recognize excellence in ways that more fully reflect the diversity of pathways through which meaningful contributions to the chemical sciences are achieved. Our intention is not to redefine excellence in the chemical sciences but rather to encourage broader reflection on how excellence can be recognized and supported in its many forms. By examining the experiences of first-generation chemists, we have determined that resilience, mentoring, community impact, flexibility, and navigating the structural challenges are some of the factors of scientific excellence that should be recognized. The following sections present insights drawn from lived experiences and professional observations within the CAS 2025 Future Leaders Top 100 scientific community. Full responses from each participant are provided in the Supporting Information.

## 1 Reconsidering Excellence in the Chemical Sciences

Reconsidering excellence in the chemical sciences first requires careful reflection on how it is currently defined and evaluated. In the most general terms, excellence refers to the quality of being outstanding or achieving the highest standards. Can we, however, measure excellence in the context of the chemical sciences? Within the context of chemical research, excellence is often evaluated through a relatively narrow set of measurable indicators, including, but not limited to, high-impact publications, citation scores, research funding, institutional prestige, and sustained research productivity.^5^ These metrics provide convenient and widely used benchmarks for evaluating scientific achievement and aspects of research performance.^5^ Nevertheless, they represent only a partial view of what we believe constitutes a complete picture of excellence in the chemical sciences. By emphasizing readily quantified outputs, these existing frameworks risk overlooking many of the intellectual, socioeconomic, and contextual factors that contribute to scientific progress.^7^ An alternative view of excellence encompasses a broader, more nuanced set of dimensions, including resilience under resource constraints, interdisciplinary thinking, mentorship, effective communication, and leadership within the scientific community. We believe these qualities not to be peripheral, rather, they are central to sustaining innovation and advancing the field. First-generation chemists often develop these skills out of necessity, by overcoming obstacles that their colleagues may not face. This exemplifies why the evaluation of excellence must be broadened.

Reconsidering excellence requires asking not only what has been achieved, but under what conditions, with what resources, and with what impact on the scientific ecosystem. These contributions, often invisible to traditional metrics, are fundamental to the progress of chemistry and the broader scientific initiative. Valuing and showcasing these journeys and contributions enables emerging first-generation chemists to see themselves reflected in the success of those who came before them, and to feel as though their own unique journeys belong in the chemical sciences. Figure 1 presents a pictorial representation of these divergent career paths and the structural challenges faced by first-generation chemists.

**Figure 1:**
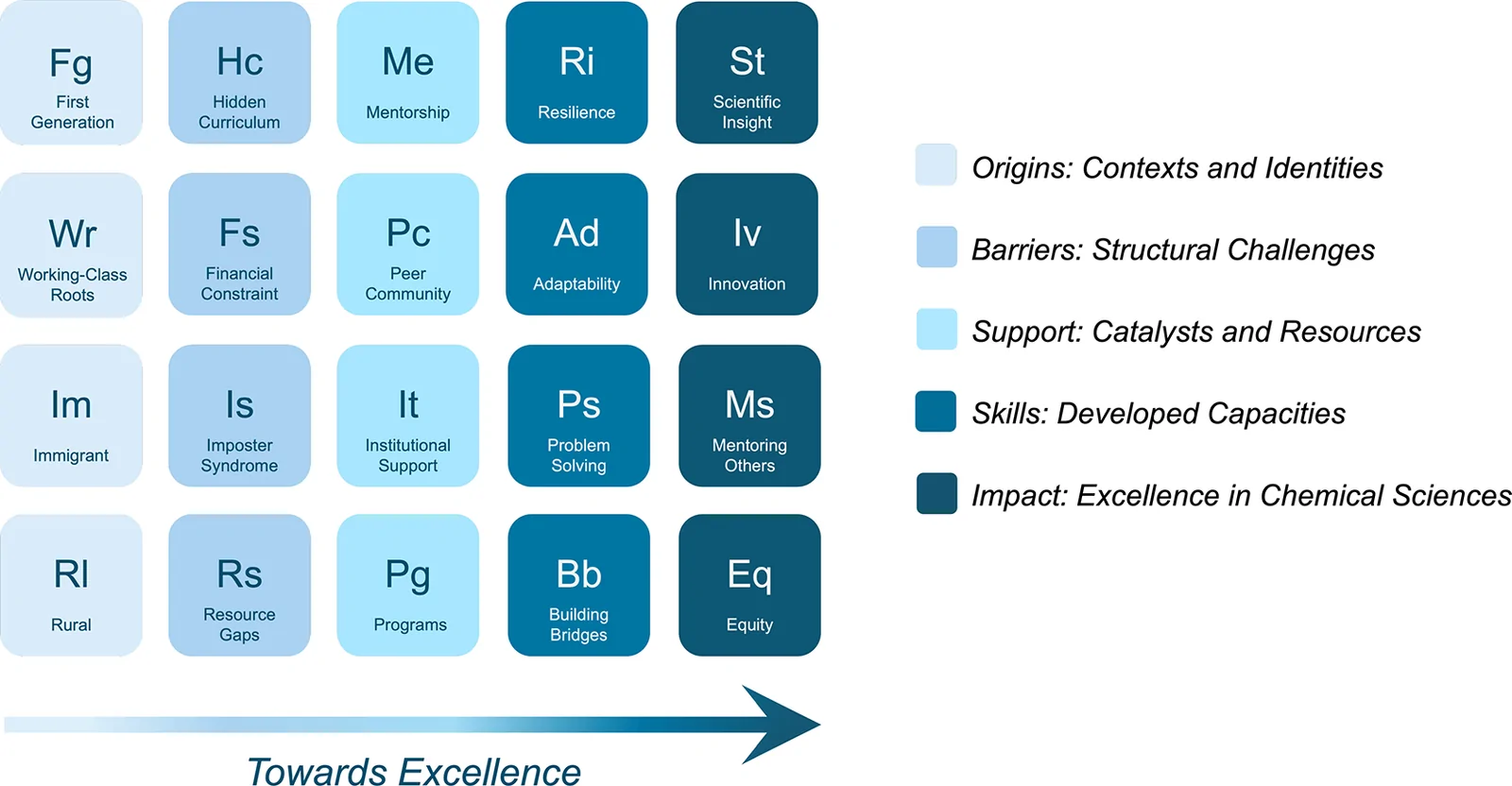
Schematic representation of the origins, barriers, support networks, skills and impact that shape the diverse career trajectories of first-generation chemists on their journey toward excellence. Figure created by Mamta Dagar and Robert A. Ives.

## 2 Pathways and Barriers for First-Generation Chemists

While many scientists encounter challenges along their professional journeys, our collective reflections suggest that first-generation chemists may face distinct barriers that can impede their progression in the chemical sciences.^8^ Three themes emerged most consistently across our individual responses (see Supporting Information). The following subsections briefly outline these key obstacles. Interestingly, the main barriers identified herein have also been found in another report in a larger population of first-generation science students, suggesting that these challenges extend beyond chemistry and are shared across the broader scientific community.^8^

### 2.1 Mentorship and Guidance

First, one of the key barriers faced by first-generation chemists is a lack of mentorship and guidance. Many first-generation chemists lack mentors and role models from similar backgrounds, reducing opportunities for relatable guidance and critical support. Without such mentors to explain the “hidden rules” of academic and industrial professions (such as the importance of networking, securing funding, being successful in applications, or career progression), first-generation chemists may be disadvantaged in navigating their careers. This is not due to a lack of ability, but largely due to limited awareness of available opportunities and pathways.

### 2.2 Financial and Economic Constraints

Financial and economic constraints represent a second major barrier for first-generation chemists. Many first-generation chemists experience disproportionate financial pressure compared with their peers (e.g., tuition, application fees, and relocation costs).^9^ These constraints can significantly shape career decision-making, at times prioritizing immediate financial stability over long-term scientific and career aspirations out of necessity. For example, these financial pressures can deter an individual from pursuing advanced training in the chemical sciences altogether.^10^ Ultimately, the journey a first-generation chemist follows may stem from the need for financial stability at that point in time, rather than their personal career ambitions.

### 2.3 Psychological Factors

An additional, yet often underrecognized factor is the psychological barrier to being a first-generation chemist. First-generation chemists, those who are the first in their immediate family to attend university, may experience a persistent sentiment of not belonging within such environments.^11^ These feelings can manifest as imposter syndrome, resulting in reduced confidence and hesitation in pursuing opportunities or advocating for oneself.^12^ This sentiment of “do I even belong here?” can hinder their ability to be visible or seize opportunities when they are presented throughout their journey in the chemical sciences.

## 3 Forms of Excellence Demonstrated by First-Generation Chemists

Excellence among first-generation chemists is frequently defined by resilience, self-direction, and the ability to navigate academic and professional environments without established guidance. Their contributions reflect not only scientific rigor but also adaptability and a strong commitment to advancing the field. Within this section, three distinct forms of excellence are identified, illustrating the diverse and impactful ways first-generation chemists shape the chemical sciences.

### 3.1 Resilience, Perseverance and Adaptability

First-generation chemists often demonstrate excellence through resilience and persistence, conveyed not only in scientific achievement but also in the capacity to adapt, self-direct, and endure despite uncertainty. In navigating unfamiliar academic systems, many describe the experience of entering academic and scientific environments without prior familiarity with institutional expectations, professional networks, or clear and articulated career pathways. This reflects a form of intellectual and professional adaptability that is distinct from what is considered a conventional form of excellence. Fostering skills of resilience, perseverance, and adaptability enables one to navigate unfamiliar settings, seek out resources, and proactively maintain progress despite structural uncertainty, giving rise to a substantive form of professional competence that contributes significantly to long-term success and stability.

This adaptability is frequently exercised under conditions of constrained resources: for example, having limited mentors in the field, a lack of financial freedom, or even available time due to family obligations. It is here that a more nuanced calibration of excellence becomes necessary. Scientific excellence is not the exclusive domain of well-resourced institutions; it emerges wherever curiosity and determination are present. Excellence can be demonstrated through high-quality and innovative research produced in the absence of strong economic or institutional support. In many cases, constraints sharpen scientific thinking rather than limit it. When every experiment must count, when reagents cannot be wasted, and when solutions must be improvised, researchers develop a precision and resourcefulness that well-resourced environments do not always demand. The ability to achieve meaningful results under such conditions reflects creativity, adaptability, and perseverance that deserve explicit recognition. Thus, evaluating scientific achievement without accounting for the resources available to produce it risks systematically undervaluing the work of researchers at under-resourced institutions. We reflect that the scientific community must move toward frameworks that ask not only what was produced, but what was required to produce it. However, we would like to emphasize that our argument does not imply that adversity is a necessary ingredient for excellence or that obstacles provide any form of advantage. Instead, these excellences arise from the abilities of many first-generation chemists to succeed in spite of such obstacles.

### 3.2 Outreach and Community Impact

A second, and equally prominent, form of excellence identified by us was the commitment to mentorship, mainly through outreach within the community, ultimately inspiring the next generation of scientists within the field. We believe that success should not be solely defined in terms of publications or awards, but in the capacity to expand access to science for others, who may not yet see themselves represented in the field, through peer mentoring, outreach activities, scientific communication, and the creation of supportive academic environments. The impact of these activities arguably strengthens retention within training programs, broadens participation, and cultivates scientific identity among emerging scholars. Collectively, these accounts suggest that excellence should be understood as a generative impact, i.e., the capacity to create opportunities, support networks, and enable pathways that allow others to enter and persist within the chemical sciences.

### 3.3 Resourcefulness and Problem-Solving Under Constraint

A third form of excellence emerges in the form of resourcefulness and creativity in addressing scientific challenges, often accompanied by a strong emphasis on real-world impact. Diverse educational and life experiences can shape how scientific problems are framed and addressed. In many cases, this includes developing the capacity to innovate under conditions of limited resources, requiring careful prioritization, practical problem-solving, and the ability to achieve meaningful outcomes under constrained means. There is also a clear emphasis on applying chemical research to societal challenges, including interdisciplinary collaboration and the design of solutions intended for use beyond the laboratory bench. Collectively, these points indicate that excellence in the chemical sciences may also be reflected in the ability to translate scientific knowledge into practical solutions and to align research efforts with broader societal needs, thereby advancing science and benefiting the public.

## 4 How Insights from First-Generation Chemists May Inform Structural Change

Having now established how barriers for first-generation chemists can lead to alternative forms of excellence that push beyond traditional metrics, we now turn our attention toward how these insights can be used to inform structural change. The lived experiences documented in this article offer a distinctive vantage point from which to evaluate existing structural supports and identify where chemistry, as a discipline, still falls short. Drawing on these insights, we highlight concrete initiatives that have demonstrated meaningful impact, examine their limitations, and propose mechanisms tailored specifically to the career trajectory of academic chemists.

### 4.1 Broadening Definitions of Excellence and Inclusion

Several responses stress the need to rethink how success in academia is defined and rewarded. This includes valuing skills such as mentorship, outreach, collaboration, interdisciplinary work, and societal impact, alongside traditional metrics.^13^ Scientific excellence should continue to be evaluated according to rigorous standards. However, funding agencies, scientific societies, and professional organizations could consider establishing awards or fellowship schemes that specifically recognize outstanding and rigorous scientific achievements accomplished in resource-limited research environments. Such initiatives would celebrate not only research quality, but also the exceptional creativity, efficiency, and perseverance often required to produce research of comparable quality under more constrained circumstances. Some institutions have begun incorporating broader impact statements into tenure and promotion dossiers, yet these efforts often remain symbolic. Reviewers and committees still default to publication count and grant money as the primary currency of evaluation, since no standardized rubric yet exists for weighing non-traditional contributions.^14^ A more innovative mechanism specific to chemistry departments would be the adoption of discipline-specific portfolio review committees, analogous to teaching portfolios already used in promotion cases. Such committees should be explicitly trained to evaluate mentorship records, method-sharing contributions, and community engagement with the same rigor currently reserved for citation metrics. Embedding this within chemistry-specific promotion and tenure guidelines, rather than relying on general institutional DEI statements, would move the recommendation from aspirational to structurally enforceable.

### 4.2 Transparency and Access to Knowledge

We emphasize the need to better communicate academic structures, expectations, and career pathways to individuals from diverse backgrounds. This includes clearly outlining routes from undergraduate study to academic careers, providing further knowledge and transparency of evaluation systems (such as publishing and funding), and reducing reliance on informal or “hidden” knowledge. Several existing initiatives address this gap at the transition into higher education and postgraduate study. For example, the Sutton Trust Summer Schools^15^ and the University of Cambridge’s Widening Access to Postgraduate Education scheme^16^ support students from disadvantaged backgrounds in accessing university and graduate programs. The ACS Bridge Program provides chemistry-specific mentorship, research experience, and financial support to ease the undergraduate-to-PhD transition. However, none of these programs addresses the ongoing discipline-specific opacity of academic chemistry progression. For instance, the process behind editorial board appointments or how PI start-up package negotiations actually work is not taught formally, even at the postdoctoral stage.

We, therefore, propose that chemistry departments and professional societies (such as ACS and RSC) develop discipline-specific transparency toolkits that cover the unwritten mechanics of academic chemistry careers and are coauthored by first-generation faculty who have navigated these pathways themselves. Improving transparency at this specific career stage is a key mechanism for leveling the playing field for first-generation scholars in chemistry.

### 4.3 Structured Mentorship and Community Support

There is a strong consensus on the importance of formalized, accessible mentorship, and support networks, rather than leaving guidance to chance or informal relationships. At the department level, peer-led orientation programs in which senior first-generation researchers guide incoming students through institutional expectations and professional norms represent an implementable step that requires no external funding or society-level coordination. Several institutions have demonstrated that more structured models can scale this further with meaningful results. For example, the University of Rochester’s First-Generation Network provides an alumni-student-family community exclusively built around first-generation identity, which offers structured mentorship and networking, integrated with career education services.^17^ Rochester’s Meliora Collective Mentorship Program^18^ has exemplified this further through one-on-one alumni-student pairings that explicitly account for first-generation background as a matching criterion, creating mentorship grounded in shared lived experiences rather than generalized professional advice. Along the same theme, Boston University’s Terrier F1RSTS Advocates Training^19^ complements the student-facing model by training faculty and staff to recognize challenges specific to first-generation students (family obligations, financial precarity, or unfamiliarity with professional norms), enabling more informed and empathetic advising.

Many of these programs share a common structural limitation. They are institution-bound; i.e., their benefits do not transfer when a first-generation student moves to a new institution for graduate school, postdoctoral training, or a faculty appointment. The University of Rochester’s model offers a partial exception by anchoring mentorship within an alumni network that persists beyond graduation. It begins to approximate the kind of cross-transition support that first-generation scholars need. We, therefore, propose an inter-institutional, society-level mentorship registry, potentially housed within ACS or RSC, that provides continuous support from undergraduate entry through faculty appointment, filling the gap that even the most well-designed single-institution models do not yet fully address.

## 5 Limitations and Scope

The insights presented are a result of the reflections of 13 first-generation chemists enrolled in the CAS Future Leaders Top 100 program. Hence, these reflections cannot represent the views of all first-generation scientists because they have come from the point of view of people who have managed to be successful in their academic activity, and have stayed within the sphere of chemical sciences. Thus, we acknowledge that this editorial has not been able to cover all first-generation scientists whose experiences have been different because some of them may have left the field of chemistry, changed disciplines, did not pursue academic careers, or were ultimately unable to overcome the barriers described.

## Conclusions

The perspectives presented across this article collectively suggest that strengthening the chemical sciences requires a broader and more nuanced understanding of excellence, one that recognizes the diverse conditions under which scientific work is conducted and the varied contributions that sustain the field. The experiences of first-generation chemists illustrate that excellence should not be defined solely by measurable outputs but also by resilience, initiative, mentorship, and the ability to create opportunity for others in the face of structural and societal constraints. Importantly, these insights do not call for abandoning established metrics but for complementing them with additional metrics that are more context-aware, inclusive, and reflective of the experiences of all individuals within the scientific environment. Ultimately, recognizing excellence in its many forms is not only a matter of fairness but a strategic imperative for sustaining scientific progress and ensuring that the next generation of chemists, regardless of background, can fully contribute to the advancement of the discipline and the broader scientific community.

## Supplementary Material



## Notes

Author biographies and full responses to questions from authors can be found in the Supporting Information (PDF)

## Abbreviations

CAS: Chemical Abstracts Service
ACS: American Chemical Society
RSC: Royal Society of Chemistry
DEI: Diversity, Equity, and Inclusion.
