# Supplementary material for: Reconsidering Excellence in the Chemical Sciences: Lessons from First-Generation Chemists
Source: ACS Cent Sci. 2026 Sep 23;12(9):1260–5. doi: 10.1021/acscentsci.6c00822 (PMC13614065; doi:10.1021/acscentsci.6c00822)
Supplement: Supplementary file 1 [file oc6c00822_si_001.pdf]

Supporting Information

Reconsidering Excellence in the Chemical Sciences: Lessons from First-Generation Chemists

Robert A. Ives,[a,*] David N. Azulay,[b,‡] Adam Cook,[c,‡] Mamta Dagar,[d,‡] Douglas J. Fansher,[e,‡] Roshan Keshari,[f,‡] Anupam Anand Ojha,[g,‡] Shivani Patel,[h,‡] Christian Sandoval-Pauker,[i,‡] Nitesh Sanghai,[j,‡] Brittany K. Trinh,[k,‡] Vishal Yadav,[l,‡] and Zhiling Zheng[m,‡]

AUTHOR ADDRESS: (a) Department of Biochemistry, University of Cambridge, Cambridge, CB2 1GA, UK; (b) School of Chemistry, Faculty of Exact Sciences, The Center for Nanoscience and Nanotechnology, The Center for Physics and Chemistry of Living Systems, Tel Aviv University, Ramat Aviv, Tel Aviv 6139001, Israel; (c) Department of Chemistry, Stanford University, Stanford, CA 94305, USA; (d) Department of Chemistry, The University of Texas at Austin, Austin, TX 78712-1224, USA; (e) (i) Department of Chemistry, Université de Montréal, 1375 Thérèse-Lavoie-Roux ave, Montréal, QC H2V 0B3, Canada; (ii) PROTEO, The Québec Network for Protein Function, Engineering and Applications, Montréal, QC G1V 0A6, Canada; (iii) CGCC, Center in Green Chemistry and Catalysis, Montreal, QC H2V 0B3, Canada; (f) Indian Institute of Technology (IIT) Bombay, Powai, Mumbai, 400 076, Maharashtra, India; (g) Center for Computational Biology and Center for Computational Mathematics, Flatiron Institute, 162 Fifth Avenue, New York, NY 10010, USA; (h) Department of Chemistry, Yale University, New Haven, CT 06520-8107, USA; (i) Smalley-Curl Institute, Rice University, Houston, TX 77005, USA; (j) College of Pharmacy, Rady Faculty of Health Science, University of Manitoba, Winnipeg, MB R3E 0T5, Canada; (k) Department of Chemistry, University of Wisconsin-Madison, Madison, WI 53706, USA; (l) Department of Chemistry, Penn State University, PA 16802, USA; (m) Department of Chemistry, Washington University, St. Louis, MO 63130, USA.

*Corresponding author: ri315@cam.ac.uk

Table of Contents

Author Biographies S2

Author Q&A S7

Author Biographies

*Corresponding author
‡Authors contributed equally to this work

Robert A. Ives (University of Cambridge)*

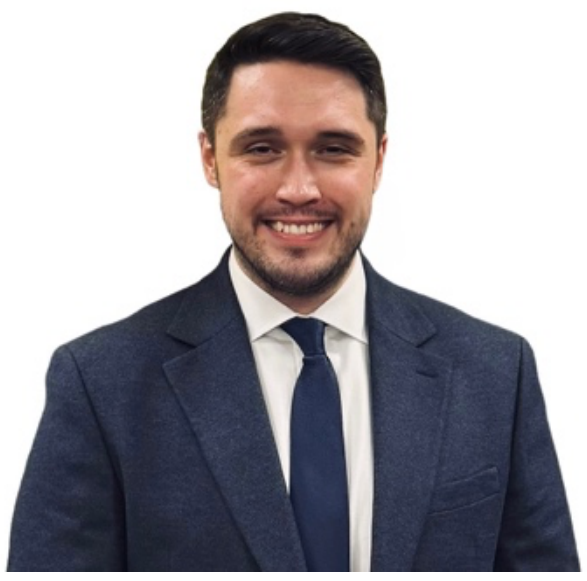

Robert Ives (b. 1997) is a British chemist who works in the fields of organic and supramolecular chemistry. Robert's training started with undergraduate (MChem, 2020) from the University of York, working under the supervision of Dr Alyssa-Jennifer Avestro, investigating organic molecular materials chemistry. During this time, he was a visiting research scholar at the Institute of Organic Chemistry and Biochemistry (IOCB) of the Czech Academy of Sciences in Prague, investigating synthetic helicene chemistry in the group of Dr Ivo Starý. He subsequently completed his doctoral studies at Durham University (PhD, 2024) under the direction of Prof Paul R. McGonigal, investigating dynamic processes in organic functional materials. He then moved to the University of Cambridge as a postdoctoral research associate in the laboratory of Dr Claudia Bonfio investigating prebiotic chemistry and lipid synthesis. At Cambridge, he is a postdoctoral affiliate of Trinity College. His contributions to science and professional service have been recognised through multiple honours, including selection as a STEM for Britain finalist (2023), the Society of Chemistry Industry Early Career Award (2025), the Yorkshire Young Achievers Foundation Award in Education (2025), and selection to the CAS Future Leaders Top 100 (2025) programme. Robert is a Member of the Royal Society of Chemistry and an Associate Fellow of Advance HE. He actively contributes to professional scientific organisations through his leadership and committee service, including roles on governance committees within the Society of Chemical Industry.

David N. Azulay (Tel Aviv University)‡

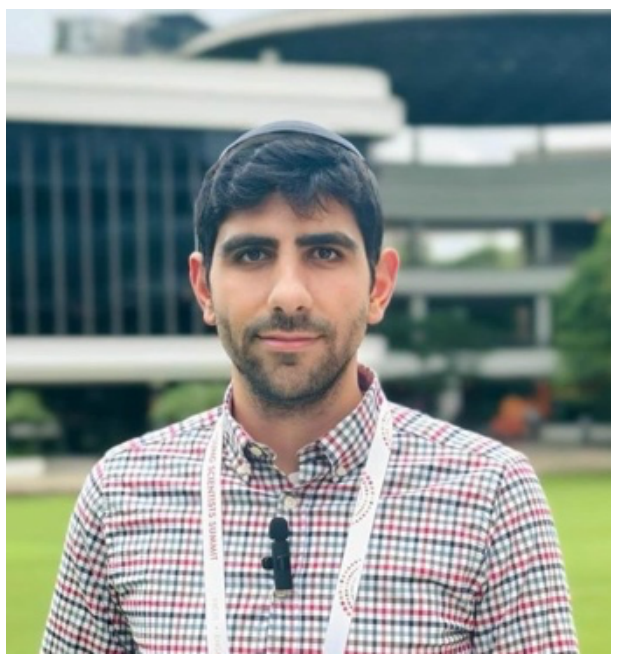

David N. Azulay (Madrid, Spain) is a postdoctoral researcher at Tel Aviv University in the laboratory of Dr. Amit Sitt, where he develops programmable, stimuli-responsive fiber and hydrogel systems for advanced shape-morphing materials. He moved to Israel to study at The Hebrew University of Jerusalem, earning his B.Sc. in Chemical and Biological Sciences and completing a direct-track Ph.D. in Chemistry under the mentorship of Prof. Liraz Chai. During his doctoral studies, David investigated the hierarchical organization and biomineralization of bacterial biofilms. His research provided key insights into bacterial biomineralization of calcium carbonate and oxalate, processes critical to biofilm integrity and kidney stone formation. Additionally, he demonstrated that the aggregation of primary biofilm proteins follows a colloidal assembly model and, using X-ray scattering techniques, elucidated the multiscale links between ions, cells, water, and biopolymers that drive biofilm development and survival. David's research and academic excellence have been recognized through numerous honors, including the 2025 CAS Future Leaders Top 100 program, selection for the 2024 Global Young Scientists Summit (GYSS), and the Israel Chemical Society (ICS) Prize for Excellent Graduate Students. Additional awards include the Nano Prize for Outstanding PhD Students, the Postdoctoral Matching Scholarship, the Kaete Klausner Scholarship, and the Faculty of Science Scholarship. Deeply committed to mentorship, he has served as a physical chemistry laboratory assistant and guided researchers from undergraduate to high school levels. Notably, his high school mentees have co-authored research in Crystal Growth & Design and received the Israel Chemical Society Peled Prize for excellence in research for work conducted under his supervision. Beyond the laboratory, he is a co-founder of the Association of Spanish Scientists in Israel, fostering scientific collaboration and visibility between Spanish and Israeli research communities. Furthermore, he is committed to community service through various volunteering initiatives.

Adam Cook (Stanford University)[‡]

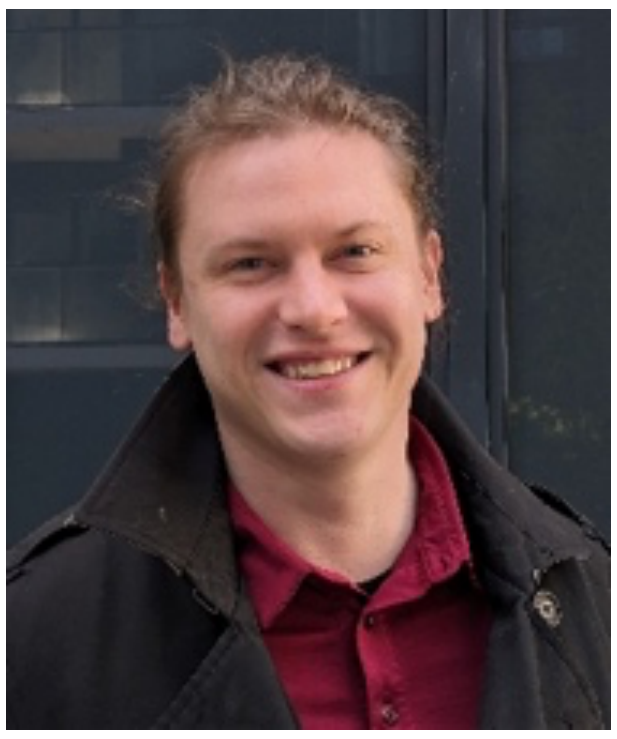

Adam was born and raised just outside of Toronto, Canada. He achieved a BSc from Ontario Tech University, working under the supervision of Prof. Yuri Bolshan, before enrolling in graduate school at the University of Ottawa in 2019 where he worked with Prof. Stephen Newman to utilize high-throughput chemical strategies to explore the world of nickel catalysis. As an exchange student, he was gracious to spend time both with Prof. Rebecca Melen at Cardiff University and with Prof. Song Lin at Cornell University. Presently, he is a Banting Postdoctoral Fellow at Stanford University where he works alongside Prof. Paul Wender on various projects related to drug delivery, HIV eradication, cancer immunotherapy and the development of RNA vaccinations. He has won >12 awards for science communication, prides himself on his ability to bring enthusiasm to the oft-mundane world of science and, outside the lab, is an avid painter.

Mamta Dagar (The University of Texas at Austin)[‡]

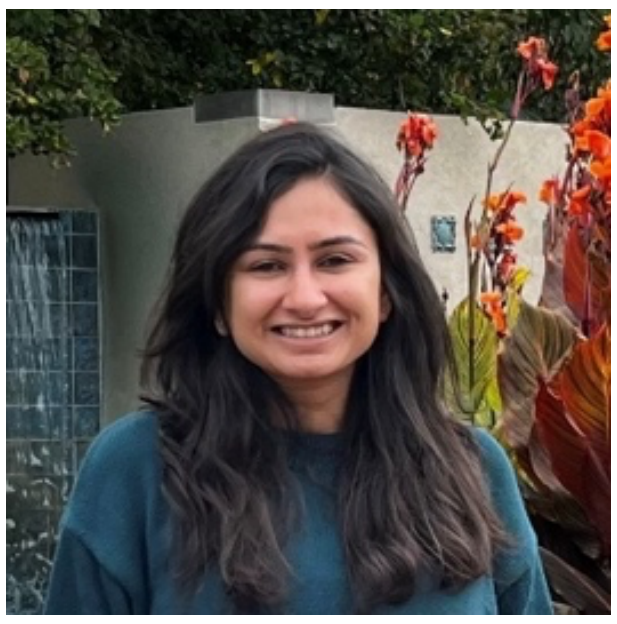

Mamta Dagar received her Ph.D. from the University of Rochester in 2025. Her dissertation focused on establishing structure-function relationships among the electrolyte components to guide the design of non-aqueous redox flow batteries. She is currently a Postdoctoral Fellow in the Department of Chemistry at The University of Texas at Austin, where she develops ion-selective electrodes and platforms for lithium extraction from brine using bipolar electrochemistry.

Douglas J. Fansher (Université de Montréal)[‡]

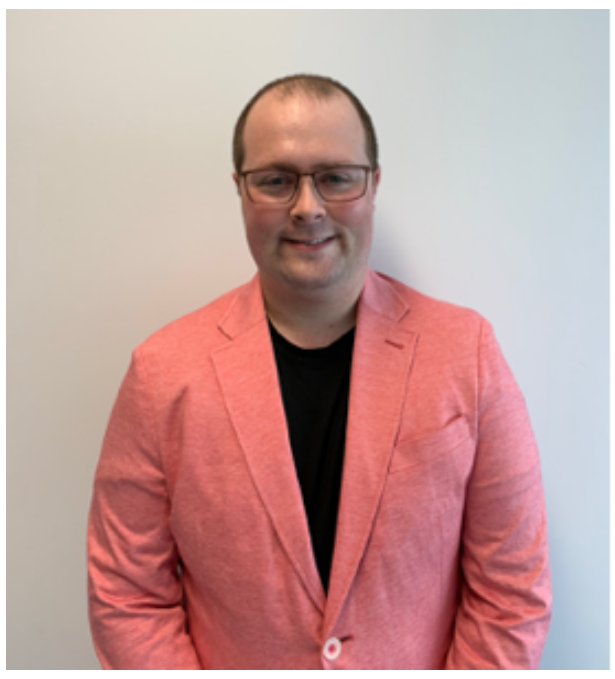

Douglas J. Fansher earned his Ph.D. in Chemistry at the University of Saskatchewan under the supervision of Prof. David R. J. Palmer, where he developed aldolases as biocatalysts for applications in organic synthesis and discovered a stereoselective enzymatic nitro-Michael reaction. He is currently an FRQNT Postdoctoral Fellow in the Department of Chemistry at the Université de Montréal with Prof. Joelle Pelletier, where his research focuses on oxidative biocatalysis, mechanistic enzymology, and high throughput engineering of cytochrome P450s. His work spans enzyme structure–function relationships, colorimetric assay development, the creation of large sequence–function datasets for predictive enzyme design, and the development of asymmetric biocatalytic reactions. His research interests include biocatalysis, reaction methodology development, and stereoselective synthesis, but he also enjoys hiking and growing cacti. D.J.F acknowledges the support of the Fonds de Recherche du Québec – Nature et Technologies.

Roshan Keshari (Indian Institute of Technology (IIT) Bombay)[‡]

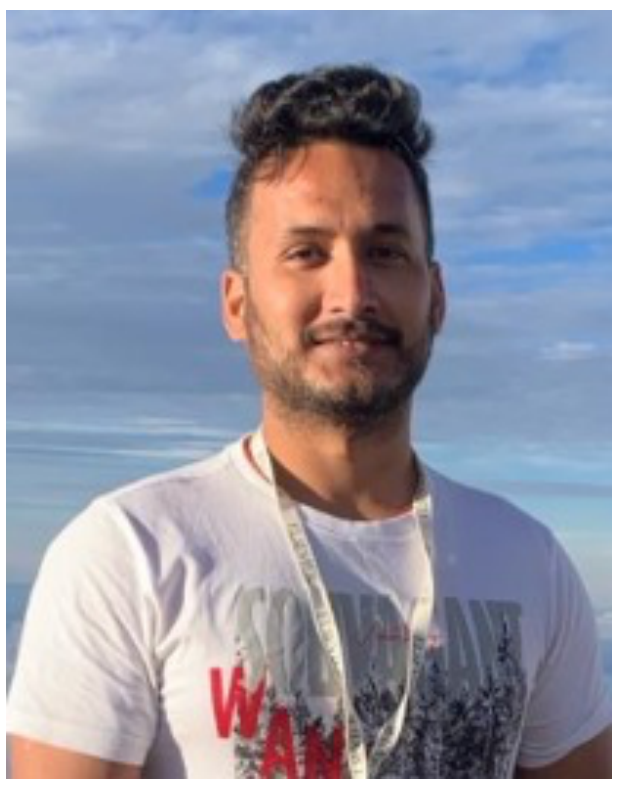

Roshan is a Biomedical Engineer with a PhD and postdoctoral experience in Biosciences and Bioengineering from the Indian Institute of Technology Bombay (IITB) and presently, as a Research Associate-II in the same department, and the co- founder of NanoTherapX Innovations Pvt. Ltd. His research integrates pharmaceutical science, nanomedicine, and bioengineering to develop translational solutions for chronic inflammatory and immune-mediated disorders, particularly psoriasis. He specialises in the design of smart, biomaterials-derived nanocarriers, lipid-based delivery systems, and hybrid hydrogels aimed at precision drug delivery, controlled release, and therapeutic monitoring. His long-term goal is to create a centre of excellence in nanotherapeutics that not only advances biomedical innovation but also prioritises access, affordability, and equity.

Anupam Anand Ojha (Flatiron Institute)‡

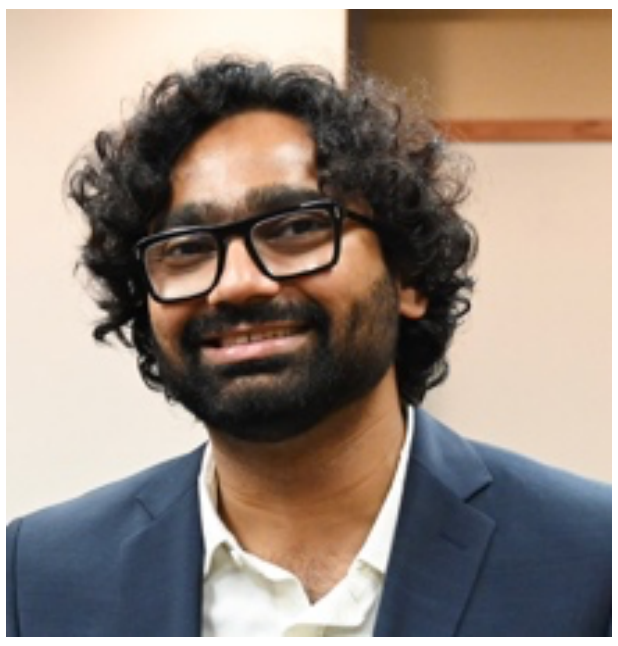

Anupam Anand Ojha is a Flatiron Research Fellow at the Center for Computational Biology and the Center for Computational Mathematics at the Flatiron Institute. He specializes in theoretical and computational biophysics, focusing on developing multiscale simulations at the intersection of cryo-electron microscopy, machine learning, and biomolecular modeling. His work aims to study protein-ligand and protein-protein interactions, model kinetic pathways involved in molecular recognition and signal transduction, and enhance understanding of complex biomolecular systems for accelerated drug discovery. Dr. Ojha earned his Ph.D. in Chemistry with a specialization in Theoretical and Computational Biophysics from University of California, San Diego, under the mentorship of Prof. Rommie E. Amaro and Prof. J. Andrew McCammon. During his doctoral research, he developed multiscale computational methods integrating Brownian dynamics, enhanced molecular dynamics, quantum mechanics, and machine learning approaches to advance drug discovery and deepen biomolecular understanding. He has made significant contributions to computational biophysics, including developing advanced algorithms and software packages such as SEEKR, QMrebind, GaMD-WE, and DeepWEST. His research achievements are complemented by a stellar academic track and a robust publication record, featuring several first-author papers in prestigious journals. He has been invited to present his work at numerous conferences, highlighting his leadership in the field. Dr. Ojha's excellence has been recognized through numerous awards and fellowships, including the Swiss Government Excellence Scholarship, Molecular Sciences Software Institute SEED Fellowship, INSPIRE Fellowship, Distinguished Graduate Student Fellowship, Teddy Traylor Award, Merkin Graduate Fellowship, Bonnie Reiss Carbon Neutrality Fellowship, Merck Research Award for Underrepresented Chemists of Color, and the Friends of the International Center Fellowship. Beyond research, Dr. Ojha is committed to equity, diversity, and inclusion. He has actively served in leadership roles such as the UC San Diego Academic Senate Committee, Chemistry International Friendship Group, and Chemistry Graduate Student Council, fostering support for underrepresented groups in science and creating inclusive academic communities. His dedication to science, mentorship, and advocacy underscores his impactful career.

Shivani Patel (Yale University)‡

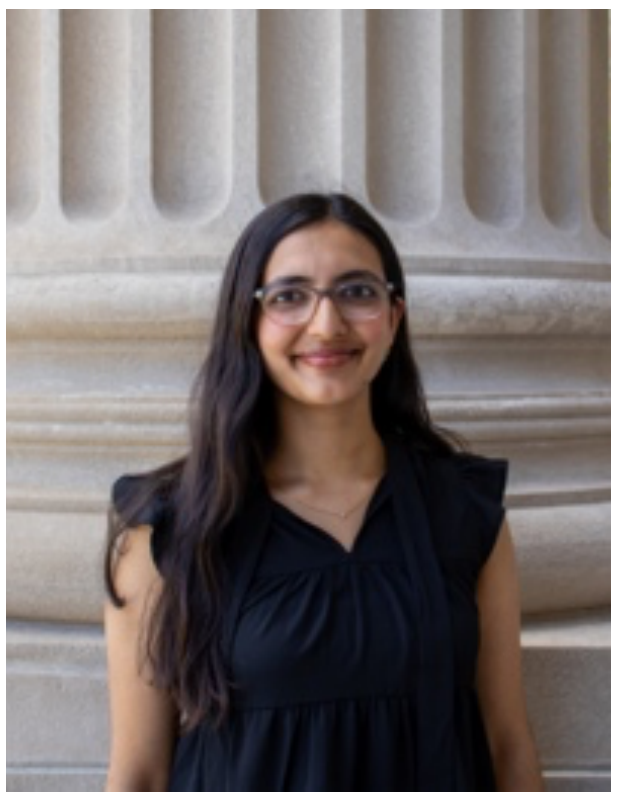

Shivani Patel is a South Asian American chemist who is a fourth year PhD candidate in the Ellman group at Yale University. Shivani first began her training in polymer chemistry as a high school student under the mentorship of Professor Frieder Jaekle at Rutgers University studying borane-modified polyolefins for use in Lewis acid catalysis. She then pursued dual Bachelor's and Master's degrees in Chemistry and Biochemistry in the Vagelos Scholars Program in the Molecular Life Sciences at the University of Pennsylvania. Under the guidance of Professor Gary Molander, Shivani investigated photochemical alkylation towards fluorinated alkanes, alkenes, and heteroarenes. She has since transitioned into her doctoral studies in Professor Jonathan Ellman's group at Yale University, where she is developing approaches to privileged high oxidation state sulfur pharmacophores by *S*-functionalization of *N*-acylsulfenamides. Over her time in the chemical sciences, she has been recognized by several honors and awards, including selection by Amgen and Johnson & Johnson Discovery Chemistry for graduate research awards, the ACS Women Chemist's Committee / Eli Lilly Travel Award, selection into the CAS Future Leaders Top 100 Program, the National Science Foundation Graduate Research Fellowship (NSF-GRFP), and the Roy and Diana Vagelos Science Scholarship. Beyond the bench, Shivani is passionate about promoting diversity, equity, and inclusion within the sciences. She has served on the board of the Mid-Atlantic Empowering Women in Organic Chemistry (EWOC) chapter since 2024 and is currently working to establish the NYConn EWOC chapter while serving as their secretary. Additionally, she enjoys mentoring talented undergraduate students through research in her lab, the Women and Gender Minorities in Science at Yale (WISAY) group, and the Yale Program to Advance Training in Health & Sciences (PATHS) program. S.P. gratefully acknowledges the NSF GRFP.

Christian Sandoval-Pauker (Rice University)‡

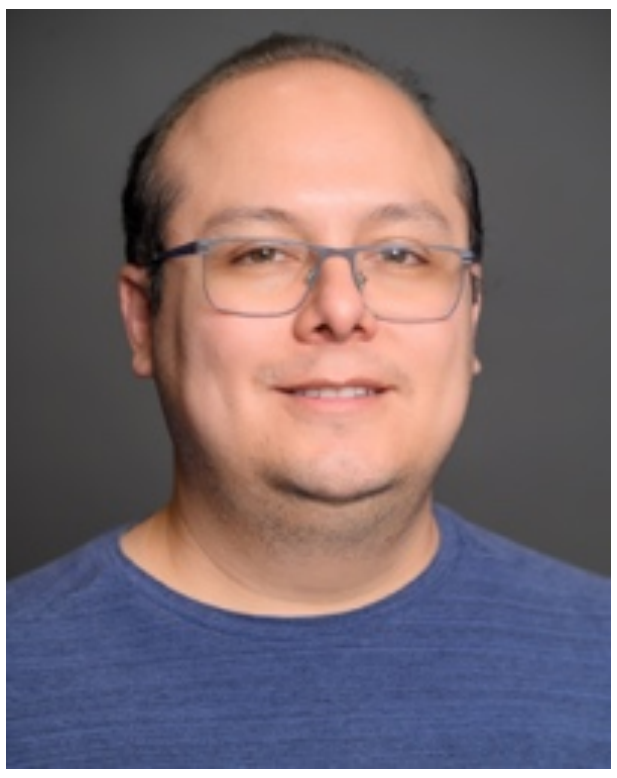

Christian received his Ph.D. in Chemistry from The University of Texas at El Paso in 2024, where he focused on computational investigations of transition metal photoredox catalysts. He earned his M.S. in Environment, Quality, and Prevention Management Systems from Universidad Internacional de la Rioja (2018) and his B.S. in Chemical Engineering from Escuela Politécnica Nacional (2016). In addition to his research training, he has experience as a university lecturer at Escuela Politécnica Nacional, where he taught General Chemistry courses and laboratories, developed course materials, and mentored undergraduate students. He joined Rice University as a J. Evans Attwell Welch Postdoctoral Fellow in August 2024, supported by the Smalley-Curl Institute. His research in the Senftle group employs electronic structure methods to advance the development of photo- and electrocatalysts for removing perfluoroalkyl substances (PFAS) from water.

Nitesh Sanghai (University of Manitoba)‡

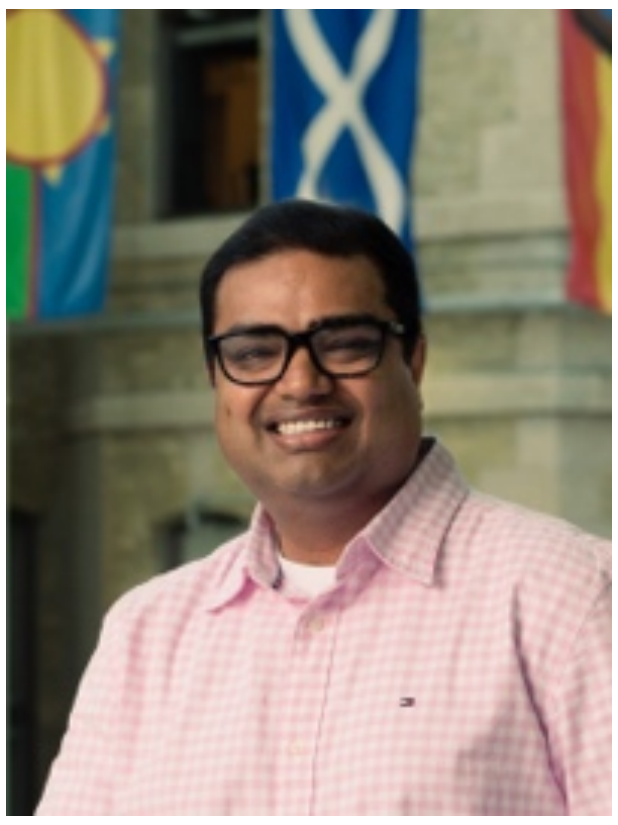

Nitesh Sanghai is a first-generation scientist and the 2025 Mitacs Innovation Award recipient for Outstanding Innovation at the University of Manitoba, Canada. He earned his PhD in Medicinal Chemistry and Neuroscience under Dr. Geoffrey K. Tranmer, during which he co-invented a first-in-class therapeutic candidate with the potential to slow the progression of incurable amyotrophic lateral sclerosis (ALS). Driven to translate discovery into impact, Sanghai and Dr. Tranmer cofounded Borotherapeutics Ltd., advancing Borsantrazole toward clinical development. He is currently a Postdoctoral Fellow in Dr. Tranmer's laboratory, focused on translating Borsantrazole from bench to the bedside of ALS patients. Committed to community-engaged research, Sanghai collaborates with ALS organizations worldwide, dedicating his career to delivering hope for patients facing this devastating disease. His work bridges science, entrepreneurship, and patient-centered innovation across disciplines globally.

Brittany K. Trinh (University of Wisconsin-Madison)‡

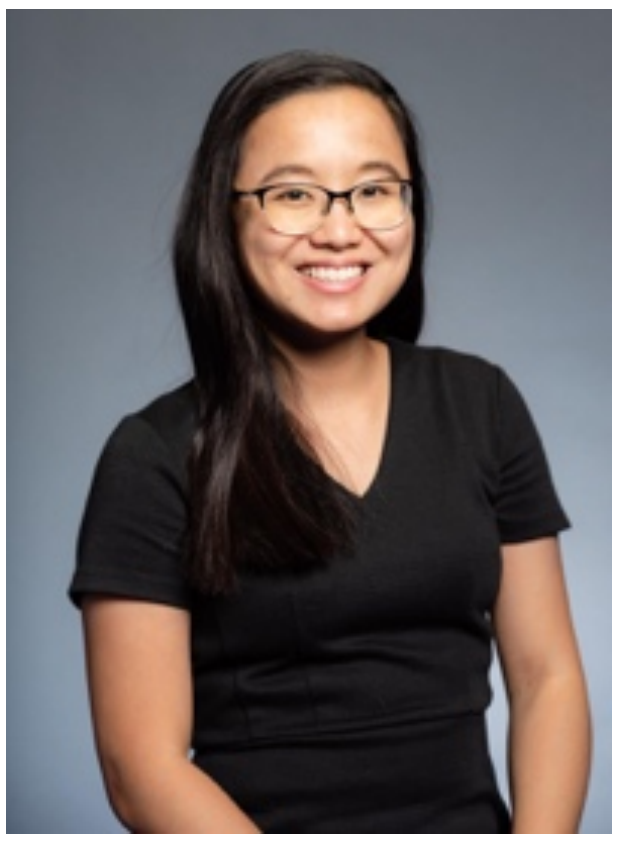

Brittany K. Trinh received her B.S. in chemistry in 2018 from the University of Houston in Houston, TX, where she worked in the labs of Prof. Debora F. Rodrigues and Prof. Melissa L. Zastrow. From 2018-2021, she worked at Sekisui Specialty Chemicals in Pasadena, TX. In November 2021, she joined the group of Prof. A. J. Boydston at the University of Wisconsin-Madison. Her current graduate research focuses on expanding applications and understanding of photoredox-mediated ring-opening metathesis polymerization.

Vishal Yadav (Penn State University)‡

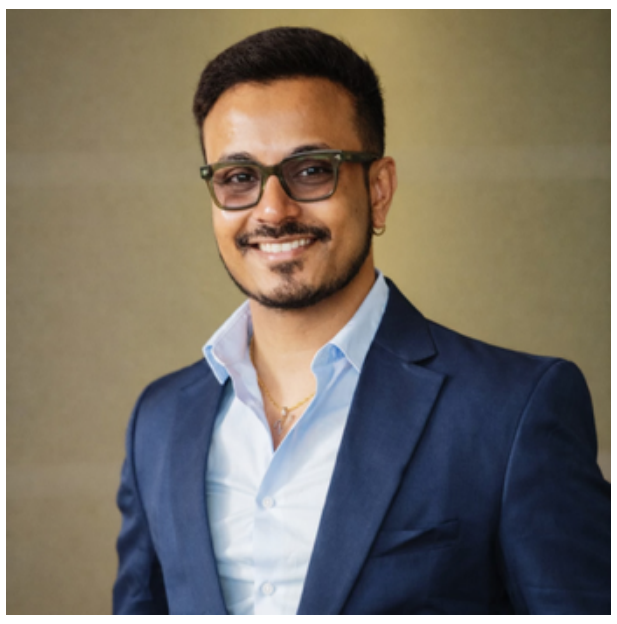

Vishal Yadav is a postdoctoral researcher in the laboratories of J. Martin Bollinger Jr. and Carsten Krebs at Pennsylvania State University. He received his Ph.D. in Chemistry from Johns Hopkins University under the supervision of David P. Goldberg, where he developed synthetic metal complexes to model enzyme active sites. His research lies at the interface of inorganic chemistry and enzymology, with a focus on understanding how metalloenzymes control radical reactivity to enable selective C–H functionalization. His current work centers on nonheme iron oxygenases and halogenases, combining spectroscopy, kinetics, and synthetic modeling to uncover mechanisms of O_2 activation and to engineer enzymes for non-native transformations, including fluorination and (iso)thiocyanation. Vishal has received several honors, including the CAS Future Leaders Top 100 (2025) and Top 35 (2026), the Merck Next Gen Leaders Award in Chemistry (2025), and the ACS PROF Leadership Development Institute Award (2025). Beyond research, he is deeply committed to mentorship and inclusive training in chemistry.

Zhiling "Zach" Zheng (Washington University in St. Louis)‡

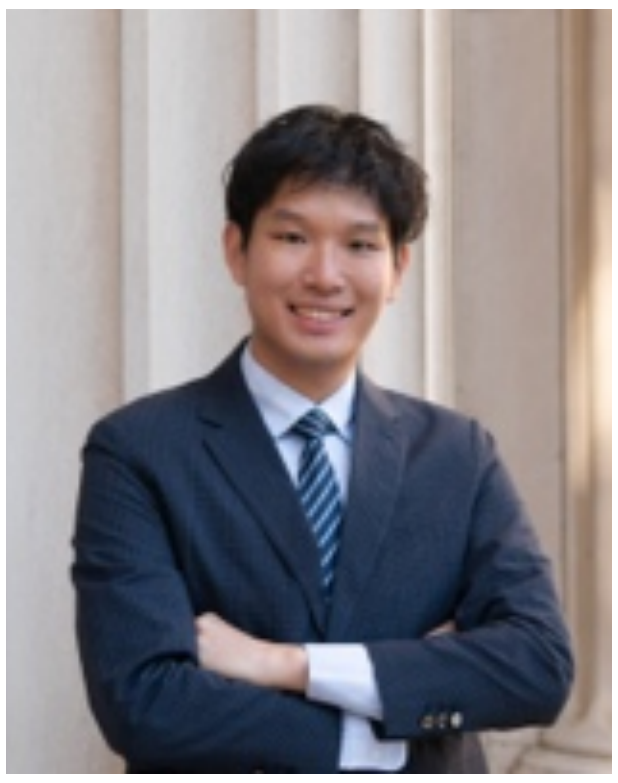

Zhiling "Zach" Zheng is an Assistant Professor of Chemistry at Washington University in St. Louis, where he leads the Deep Synthesis Lab, integrating AI with chemical synthesis to accelerate the discovery of crystalline porous materials. He began his academic journey at Cornell University, conducting undergraduate research with Prof. Kyle Lancaster and earning a Bachelor of Arts degree in 2019. He then moved to University of California, Berkeley, where he completed his Ph.D. in Chemistry under the supervision of Prof. Omar M. Yaghi, focusing on metal–organic frameworks for atmospheric water harvesting. He carried out postdoctoral research in Chemical Engineering at Massachusetts Institute of Technology with Prof. Klavs F. Jensen in 2023–2024. Dr. Zheng's work has been recognized with the 2025 Carbon Future Young Investigator Award and the Inflection Award for AI-driven climate solutions. He was also a finalist for the Dream Chemistry Award and a runner-up for the Chen Institute and Science Prize for AI Accelerated Research.

Author Q&A

1. What is meant by excellence in the chemical sciences, and what reasons might there be to re-assess this concept?

David: Excellence in the chemical sciences is typically assessed through visible and quantifiable achievements: high-impact publications, competitive funding, institutional recognition, and sustained research productivity. Although these metrics are important, I believe excellence should be reconsidered in broader and more meaningful terms. First, excellence is originality. It is the capacity to ask new questions, to approach problems from unconventional angles, and to generate ideas that move a field forward. This kind of originality is not necessarily correlated with institutional advantages or access to extensive resources. Second, excellence can be demonstrated by producing high-quality and innovative research even in the absence of strong economic or institutional support. The ability to achieve meaningful results with limited resources reflects creativity, adaptability, and determination. In many cases, constraints can sharpen scientific thinking rather than limit it. Third, excellence stems from passion and resilience. Scientific research is inherently uncertain and often slow. The capacity to persist through setbacks, to remain motivated, and to continue refining ideas despite obstacles is a fundamental dimension of scientific excellence that is not always captured by traditional evaluation systems. Finally, an excellent scientist is not only someone who makes discoveries, but someone who knows how to communicate them. Transmitting scientific knowledge beyond the immediate research community, to students, to the public, and to society at large, strengthens science as a whole. When knowledge reaches broader audiences, it does not only advance a specific subfield, it contributes to the progress of science and, ultimately, to humanity. Reconsidering excellence in these terms allows us to recognize forms of merit that are sometimes overlooked by conventional assessment frameworks.

Mamta: In academia, excellence is often assessed by a set of metrics such as grades, test scores, publications, fellowships, and the prestige of institutions. These assessment indicators overlook the equally important qualities that shape a scientist: resilience and the personal struggles navigated along the way. For many first-generation chemists, the path into and through science is marked by financial constraints, cultural and familial expectations, and a constant need to self-advocate in systems they were never explicitly taught to navigate. When we treat “excellence” as a single umbrella label and ignore these individual journeys, we not only flatten people’s stories but also reinforce a narrow, exclusive picture of who “deserves” to be called excellent. A more humane and meaningful perspective is to see humble beginnings, detours, and obstacles as integral parts of the scientific journey, not as footnotes to an impressive resume. Naming and valuing these trajectories can be transformative for budding chemists from similar backgrounds, who might finally recognize themselves in the lives of first-generation scientists who came before them and feel that their own paths, however non-linear, also belong in chemistry.

Douglas: Chemistry and other scientific fields are still defined by a small group of criteria, such as starting a career in research early on, publishing often and in important journals, and knowing the right people in the scientific community. For a scientist who's just starting out, everything must be learned, so being excellent looks different for everyone. It can be hard to navigate this landscape without a plan. A more meaningful definition of excellence that would be more widely applicable would recognize strengths related to the process of achieving a goal, including *how* you do what you do, not just the *result* of what you do. In other words, it would recognize the journey, not *just* the destination. A person's ability to build structure, mentor others, design efficient workflows, and create a clear path in a complex environment are core scientific competencies that are not necessarily equally considered. For people who aren't very familiar with science, the journey itself can be just as meaningful as the destination.

Roshan: As a first-generation scientist, my understanding of excellence in the chemical sciences has evolved through personal experience rather than inherited familiarity with academic systems. Early in my career, excellence seemed defined primarily by publications, journal impact factors, and grant funding. While these measures are important, my journey in nanomedicine research and technology translation has shown me that excellence also lies in transforming scientific knowledge into meaningful societal impact. Without a family background in science, many aspects of research culture, such as navigating fellowships, publishing strategies, intellectual property, and research funding, were unfamiliar and had to be learned independently. This experience shaped my perspective that excellence is not only about scientific output but also about persistence, self-direction, and the ability to create opportunities despite limited guidance. My work in developing nanotechnology-based therapies reinforced the idea that impactful science often extends beyond academic recognition. Translating laboratory discoveries into accessible healthcare solutions required interdisciplinary collaboration, problem-solving under resource constraints, and long-term commitment. These efforts are rarely captured by traditional metrics, yet they represent meaningful contributions to the chemical sciences. Reconsidering excellence in the chemical sciences, therefore, requires acknowledging diverse pathways into research and valuing contributions that extend beyond publications. A more inclusive understanding of excellence would recognise translational impact, innovation, mentorship, and the resilience often demonstrated by first-generation scientists. Broadening this definition would better reflect the realities of modern chemical research and encourage a more equitable and impactful scientific community.

Anupam: The current definition of excellence in the chemical sciences relies heavily on quantitative metrics, including but not limited to publication output, citation impact, and institutional affiliation. While these indicators capture aspects of research productivity, they fail to account for the full range of capacities that sustain scientific communities and drive long-term innovation. Resilience under resource constraints, cross-cultural communication, interdisciplinary thinking, and community leadership are not peripheral to scientific excellence, but they are constitutive of it. First-generation scientists, who often develop these capacities out of necessity, offer a compelling case for broadening the field's evaluation frameworks. Reconsidering excellence means asking not only what has been produced, but under what conditions, with what access to resources, and with what broader impact on the scientific ecosystem.

Shivani: Excellence in the chemical sciences was pre-defined in a period where there was a lack of diversity in communities able to access science. As time goes on, it is increasingly important to reconsider what we deem as "excellence" given the ever-changing circumstances of the world and landscape of individuals wanting to be involved. Recognizing excellence is important because it allows those who work to advance scientific knowledge to be recognized and progress in their careers. It is inspirational to colleagues and peers alike by incentivizing good practices. For example, as more BIPOC community members are seeking to become chemists, are they marginalized in being recognized for their excellence? Are first-generation scientists, due to various factors I have discussed in the following sections, not being recognized? Additionally, can one measure excellence in an area other than scientific progress of research? In a fast-moving field with new chemistries being constantly developed, can we look beyond the science for "scientific progress"? Could excellence look like an individual pioneering programs to improve diversity, equity, inclusion, and accessibility for example?

Christian: To understand how we can reconsider excellence in the chemical sciences, we first need a clear definition of what excellence is. A textbook definition of excellence is being notably superior and consistently meeting the highest standards. However, how can we measure excellence in the context of chemical sciences? Are the existing quantitative metrics, such as the number of papers published or citation metrics, a fair representation of excellence in chemistry? Or are there other forms of excellence we are ignoring? There are many voices in chemistry, and all of them are shaping the future of the world. Some of those voices are focused on generating discoveries, others on addressing direct needs in their own communities, and others on making an impact on industry or advocating for a more inclusive and sustainable world. We also cannot forget the thousands of educators and science communicators who are encouraging the next generation of chemists, including first-generation chemists like us, to pursue careers in the chemical sciences. Given the many actors in chemistry, it feels impossible and biased to rely on the same metrics for all of them. It is also difficult to create a quantitative metric of excellence that accounts for the different realities in which all these actors operate. At the end of the day, excellence is subjective, maybe even personal. If we go back to its definition, we are all excellent in one way or another because we are pushing the boundaries and trying to build a better society every day.

Brittany: There are many students who are excellent in other aspects of science beyond the number of publications, such as mentoring/training abilities, technical communication/presentation skills to non-experts, troubleshooting skills, flexibility/adaptability, building community, providing professional development support, and more. As a 5th year graduate student who currently has no publications (due to projects changing), I have to remind myself that there are other scientific skills that I have that I am good at, and publications are not the only measure of excellence in the chemical sciences, at least in academia. I've found that looking at industry standards and considering the other skills and traits they value, has helped me improve my confidence in my abilities as a scientist. For example, since I changed projects midway my PhD, from a polymer characterization project to a more synthetic polymer chemistry project, I've picked up on a wider variety of skills. Although I am not the expert or best at all of these skills, my ability to be flexible and adapt quickly would be valued in industry, where it is advantageous to be a quick learner, especially if the company's technologies/products/projects/priorities change often. Additionally, I have mentored/trained undergrads, graduate students, and even senior hires for a startup. This experience may not be documented in awards or directly in my CV, but the ability to discuss science and successfully work and communicate with people at different levels of knowledge and experience should not be overlooked. Even within our own department, we have awards for mentors, but there are plenty of mentors who are doing a good job, but simply are not recognized properly.

Vishal: My experience in academia has made me reflect on how narrowly "excellence/success" is often defined in the chemical sciences. Metrics such as publication counts, journal impact factors, institutional pedigree, and funding success are frequently used as proxies for excellence. While these measures capture some aspects of scientific productivity, they often overlook the broader qualities that make a scientist impactful: creativity, resilience, mentorship, collaboration, and the ability to build new interdisciplinary bridges. As someone who trained across different countries and institutions, I have seen how structural advantages, such as access to networks, early research exposure, or familiarity with academic norms, can strongly influence these conventional metrics. Excellence is therefore not always a neutral measurement of ability; it can also reflect accumulated opportunity. I believe the chemical sciences would benefit from a broader framework for evaluating excellence, one that includes contributions to mentorship, scientific community building, interdisciplinary thinking, and the ability to navigate complex intellectual and practical challenges. Many transformative discoveries arise from unconventional trajectories, and recognizing these paths is essential for sustaining innovation in the field.

Zhiling: My view of excellence shifted from "good at lab work" to "turn curiosity into real-world impact." I started crystallization in my kitchen as a child (salt, alum, rock candy) before I ever saw a lab. Now, I see excellence as combining rigorous chemistry with new tools to solve urgent problems like atmospheric water harvesting and carbon capture.

2. What are the pathways and barriers for first-generation chemists?

David: For first-generation chemists, the entire academic system is new territory. There is no inherited map explaining how universities function, what to prioritize at each stage, or how to balance coursework, research, and long-term career planning. Much of the path must be discovered independently. In my case, my interest in science began long before I understood what a scientific career entailed. It started with a deep admiration for nature, nurtured by a children's magazine on science and technology that I received for several years. I still remember how much I loved reading it the first time. That sustained exposure gradually transformed fascination into curiosity. At home, that curiosity often translated into hands-on experimentation. I spent hours exploring and recreating scientific experiments, including a simple pH detector using red cabbage, a recreation of the double-slit experiment, and a small Franklin bell. I was also influenced by science communicators and educational television programs that made scientific discovery feel both exciting and accessible. My parents are not scientists and work in completely different fields. Yet they played a fundamental role in shaping my trajectory. My mother would give me experiment kits to explore at home and consistently emphasized the importance of higher education as a gateway to opportunity. My father, although not scientifically trained, always highlighted the importance of reading and enthusiastically shared news about scientific discoveries. They worked hard to provide me with opportunities they themselves had not had, and they remain deeply proud of my path, even if the technical details of my work remain largely unfamiliar to them. However, entering academia revealed a different layer of complexity. The scientific career path, from undergraduate studies to PhD, postdoctoral training, and potentially professorship, is rarely explained explicitly. Without family familiarity with this structure, it can feel opaque and uncertain. Questions about stability, remuneration, and long-term prospects are constantly raised by those around me. There is also limited understanding of what it means to be a doctoral student or a postdoctoral researcher. I have frequently been asked when I will "start a real job," reflecting a broader societal misunderstanding of scientific work. There is often an implicit assumption that science is generational. When I mention that I am pursuing a postdoctoral career, I am frequently asked whether my parents are scientists as well, as if belonging to the scientific community were something inherited rather than constructed. Being new to the academic system, without family members who have navigated it before, can create a persistent sense of being an outsider. This feeling can, at times, contribute to impostor syndrome. For first-generation chemists, the pathway is therefore shaped by both deep personal motivation and the challenge of navigating an unfamiliar system without a guide.

Mamta: Growing up in the Indian school system, I felt the weight of choosing a "stream" right after 10th grade without really understanding what any of those choices meant for my future. While many of my peers seemed to have had their paths mapped out for years, preparing methodically for engineering or medical entrances, I often felt a step behind, unsure of what I truly wanted and intimidated by their certainty. At home, the message was clear: a practical, respectable path meant becoming either a doctor or an engineer, not a researcher, and certainly not a chemist traveling far from home. It took persistent, emotionally exhausting conversations just to be allowed to commute as a day scholar to a nearby city for my Bachelor's degree, and even more negotiation to move to the opposite end of India for my Master's program. When the possibility of going to the USA for a PhD emerged, the resistance was so strong that I almost abandoned the idea altogether. In that moment, my Master's PI's simple offer "Tell your father it is a great opportunity for you, and if he doesn't agree, I don't mind talking to him personally" felt like a lifeline. That validation gave me the courage to push once more, and slowly my parents began to accept, even if traces of their reservations still surface in conversations. Looking back, those tensions between familial expectations, cultural ideas of "safe" careers, and my own emerging desire for a research life in chemistry have shaped not only my academic path but also the way I now think about enabling and constraining forces in first-generation journeys.

Douglas: My own journey reflects some common challenges. I entered university without the knowledge that many of my peers possessed who are not first-generation scientists. I first wanted to be a doctor. I didn't know about the process to become a scientist and I also didn't know that it was a good choice for me. It wasn't until the last year of my undergraduate studies, during my honours research thesis project, that I realized that research was something that I truly enjoyed and wanted to do. At that point, my career path changed. I think this is somewhat common for first-generation scientists because it's hard to know you want to do something that you've never been exposed to. My dad was an industrial mechanic and welder, while my mom was a stay-at-home mom who managed our farm. Science was not something we talked about in our day-to-day lives. My undergraduate, PhD, and postdoc mentors helped me a lot as they made the scientific system clearer and explicitly stated the expectations present in all areas of research and science. These people are great at mentoring. They had a direct impact on my success in receiving the CAS Future Leaders (Top 100) award and are why I enjoy student training/mentorship and research.

Roshan: My pathway into the chemical sciences was shaped largely by curiosity and persistence rather than a clear roadmap. As a first-generation scientist, I entered research without prior exposure to academic career structures, which meant that many important decisions, such as selecting research directions, identifying funding opportunities, and planning long-term career goals, had to be navigated independently. Progress often depended on seeking information proactively and learning through experience rather than relying on established networks or informal guidance. One of the major barriers I encountered was the lack of early awareness about research careers and the opportunities available within the chemical sciences. Many first-generation students discover research relatively late, which can place them at a disadvantage compared with peers who have early exposure to scientific environments. Limited access to professional networks and mentors can also make it more difficult to identify opportunities such as fellowships, international collaborations, or interdisciplinary projects. Financial considerations can also shape the pathways of first-generation scientists. The need for stability often influences career choices, and pursuing long-term research goals or entrepreneurial activities can involve significant uncertainty. In my own experience, balancing academic research with translational work and startup development required careful management of both time and resources. Despite these challenges, several enabling factors played an important role. Supportive mentors who provided guidance beyond technical supervision helped bridge knowledge gaps and build confidence. Exposure to interdisciplinary research environments created opportunities to develop broader perspectives and practical problem-solving skills. Recognition through awards and fellowships also helped increase visibility and opened doors to collaborations. Overall, the pathways of first-generation chemists are often characterised by self-directed learning and gradual accumulation of opportunities. Reducing structural barriers through mentorship, clearer career guidance, and accessible professional networks would help ensure that talented first-generation scientists can fully contribute to the chemical sciences.

Anupam: First-generation chemists navigate a set of structural barriers that are not individual in origin. These include limited access to preparatory resources, absence of professional networks, language and cultural displacement upon entering academic institutions, and a persistent lack of mentorship from individuals with shared backgrounds. Financially, the cost of exam preparation, relocation, and early-career research participation falls disproportionately on students from low-income backgrounds. Socially, the transition into institutions with different linguistic and cultural norms can undermine confidence and a sense of belonging at precisely the stage when scientific identity is being formed. What enables persistence, where it occurs, is typically a combination of targeted institutional support, including scholarships, structured mentorship programs, and inclusive student organizations, alongside informal community-building efforts often led by students themselves. The evidence suggests that retention improves when support is proactive and embedded in institutional structures, rather than dependent on individual initiative or chance.

Shivani: On mentorship as a critical resource for pathways to chemistry: Growing up and watching my family help at the family store, I always said proudly that I would love to work there one day. I accepted happily that this would be my destiny. Then, I realized that I enjoyed chemistry in high school, where I had a teacher, Mr. Stanton, who inspired me to want to learn more about "molecule bending and breaking". As the first chemist in my family, I did not know how I could investigate my curiosities more seriously. Participating in the high school science fair, there weren't many things that I could pursue that could be safe in a classroom setting. I asked Mr. Stanton if there was any way I could do research "somewhere else" just to see if I liked it. I had no idea what university-level research looked like, but my curiosity paired with a passionate and kind high school teacher landed me in the lab of Professor Frieder Jaekle at Rutgers University, Newark. This is where everything changed for me. Professor Jaekle was a wonderful mentor who encouraged me to get my hands wet in the development of triarylborane polymers as supported catalysts for Lewis acid-catalyzed hydrosilylation. Having the courage to ask for opportunities was critical for me as a first-generation chemist but also having mentors who went above and beyond and advocated for me opened up my pathway to chemistry. It allows for the barrier of inclusion to be lowered and barriers to be crossed more easily, with these mentors becoming "pseudo" family in the casual conversations and advice shared. Finding career guidance is an inherent barrier for first-generation chemists, where they often must seek out this information, which I have often felt like I have had to.

On financial burden as a barrier to chemistry: As a first-generation chemist, I am also the first in my family to go to graduate school. My parents did not imagine or have the opportunity to go to graduate school since they had to work to build a life for me and take care of their parents. While I am incredibly grateful that going to graduate school was an opportunity available to me, for myself and I think for many other first-generation chemists, the financial instability that could come with going to graduate school and pursuing a career out of passion rather than financial drive is stressful. Many of my first-generation friends and I have talked about the long-term financial impact that could come from going to graduate school and essentially starting a real career at 30. Since we are not paid a lot, I can understand how some first-generation chemists that have immense financial pressures may not even have the option to pursue a graduate degree.

On the mental health impacts as a barrier to chemistry: In addition to academic stressors, many first-generation students have financial concerns and family obligations. Personally, I feel as though these additional stressors that first-generation students face is more likely to put them at risk for mental health challenges. In addition to these compounding non-academic obligations, in a specialized field such as chemistry, the lack of diversity can negatively impact these students. As a South Asian woman, there aren't a lot of people who look like me in this field. At times, this has led me to feel imposter syndrome and lose a sense of belonging. This is a significant barrier within chemistry that can cause lack of retention of first-

generation chemists from the BIPOC community. The presence of allies that advocate for these individuals could dramatically impact self-confidence and career opportunities for first-generation peers.

Christian: Over the last few years, we have seen exponential growth in competition for academic research funding. For instance, during the last call of the 2025 Marie Skłodowska-Curie Actions (MSCA) Postdoctoral Fellowships, the number of applicants increased nearly 65% compared with 2024, exceeding 17000 with a success rate of ~10%[1]. In the US, the story is similar, with universities seeing larger cuts in research funding.[2] In this highly competitive environment, many first-generation chemists face several barriers, including limited information, mobility challenges, difficulty accessing permanent jobs or alternative career pathways, and work-life balance issues. Many first-generation chemists are international students migrating from their home countries to more developed countries in search of educational opportunities. However, restrictive visa regimes, high costs, and the inequalities faced by scholars from developing countries are profound challenges for first-generation chemists when traveling abroad. The lack of knowledge of how the system works and limited connections make the paths of first-generation scientists more complex and uncertain. Resilience, time, and effort are therefore required to overcome those limitations, which could slow down their career progression compared to other peers. Beyond these structural and mobility challenges, broader systemic pressures within academia further shape the experiences of first-generation chemists. Work–life balance in today's academia is a growing concern. The current lack of funding and support is pressuring researchers to finish projects and publish more quickly, while the resources to do so are decreasing.[3] In this environment, a first-generation chemist is obliged to adapt even faster. Access to a job after PhD training is also a challenge that all doctoral students face. Here again, visa constraints, a lack of a broad professional network, and limited exposure to non-academic career pathways are among the barriers that first-generation chemists face. In academia, short-term contracts and funding uncertainty complicate long-term planning. Addressing these barriers will require coordinated institutional efforts to expand career development resources, improve access to mentorship and networking opportunities, increase transparency around career pathways, and provide stronger support mechanisms for first-generation international scholars, including visa guidance and transition support.

Brittany: As a first-generation college student, I had no idea about graduate school until I serendipitously learned about it through stumbling into undergraduate research opportunities. I was struggling in my engineering class and wanted to get some hands-on experience, so I reached out to the course instructor, who introduced me to a grad student in a lab, who introduced me to the professor of the lab. From this experience, I was able to apply to undergraduate research fellowships at the university, and get paid to do research. Being paid to do research was beneficial to me, as I paid my tuition by myself due to my family's financial situation at the time. It would have been difficult to pursue research otherwise. The CHOPs program at UW-Madison (https://pubs-acs-org.ezproxy.library.wisc.edu/doi/10.1021/acs.jchemed.1c00528) is also one of the reasons why I am in graduate school. The program helped me understand what the application process and graduate school experience entailed. It was a fully paid experience for me to learn more about the chemistry department at UW-Madison before even applying to the university. At the end of the program, they gave me a fee waiver to apply to the program. Knowing that UW-Madison was a top 10 university in chemistry, I was unsure about my chances of being accepted since my GPA was considered low for graduate programs (3.36 out of 4.0 GPA). However, since I received a waiver, I decided to apply just to see if I could get in. To my surprise, I was accepted and offered an additional fellowship. Without the CHOPs program and the waiver, I probably would not have had the confidence to apply and would not have pursued a PhD in chemistry. I also grew up fearing debt. Even though I learned from professors that I would get paid a stipend in graduate school, I wanted to be financially stable prior to going to graduate school because I did not want to worry my family about the finances of moving or having to deal with any emergency situations. I was fortunate enough to get a position as a chemist in a technical service position after graduating (while deferring my acceptance to UW-Madison). This experience also helped inform my research interests before graduate school. Although my family does not understand what I do in my research, they are supportive of my educational goals. When I talk to prospective graduate students about graduate school, I also suggest to them to consider this aspect of pursuing a PhD as well. Another barrier I've noticed is due to social or cultural differences. As a Vietnamese-American, I was raised to respect elders and "not talk back". Although this is helpful when it comes to elders in my culture, it is not necessarily as helpful in an academic environment, where some professors consider you more as a colleague and insist on you calling them by their first name. They probably expect that you will push back occasionally and disagree on research approaches. For first-generation students, including myself, I observed that we felt inclined to implement every suggestion our professor gave, even if it didn't make sense sometimes. More senior graduate students encouraged me to consider suggestions more critically and to not be afraid to disagree or speak my mind directly to my professor. Over time, I realized that this wasn't talking back, but it was part of managing expectations and priorities, or "managing up." I've noticed that for other first-generation students, it took them longer to realize this, so they struggled more with managing up. Perhaps it would be helpful for professors to be more cognizant of these social or cultural differences when new lab members join, so that they can slowly adapt to different communication and work styles.

Vishal: First-generation scientists often enter academia without the implicit knowledge that others may inherit through family or social networks. Many of us learn the "rules of the game" only after encountering them, whether those rules relate to applying for fellowships, building professional networks, or navigating institutional hierarchies.

Some barriers I have observed include:

- Limited early exposure to research careers, which can delay entry into competitive research opportunities.
- Lack of informal mentorship, particularly guidance on unwritten norms such as networking, negotiating positions, or building collaborations.
- Financial and geographic constraints, which can limit opportunities to attend conferences or pursue research experiences.
- Psychological barriers, including imposter syndrome and uncertainty about belonging in academic spaces. At the same time, there are enabling factors that can significantly change trajectories. Access to strong mentors, supportive research groups, and inclusive scientific communities can help bridge these gaps. Programs that provide structured mentorship, travel support, and transparent information about academic pathways can be particularly transformative.

Zhiling: As a first-generation student, I faced financial pressure, limited guidance on the "hidden rules" of academia, lack of networks, and periods of self-doubt. First-generation student organizations (with peer advisors) in my undergrad school and summer research opportunities offered by my undergrad department were key supports that helped me persist and reach graduate school.

3. What are the forms of excellence demonstrated by first-generation chemists?

David: First-generation chemists often demonstrate forms of excellence that are not always immediately visible within traditional academic metrics. One such form is intrinsic motivation. For many first-generation scientists, entering academia is not the continuation of a family tradition but a personal decision driven by vocation and curiosity. The choice to pursue science emerges from genuine fascination rather than inherited expectation. This often translates into deep commitment, perseverance, and long-term dedication. Another distinctive strength is resilience. Navigating an unfamiliar system without inherited academic guidance requires adaptability and self-direction. Learning the unwritten rules of academia independently fosters autonomy and determination. The ability to persist despite uncertainty, external questioning, or structural opacity becomes, in itself, a form of excellence. First-generation chemists may also bring out of the box thinking. Approaching science without strong preconceptions shaped by academic lineage can encourage fresh perspectives and unconventional problem solving. Discovering the scientific world as something new can preserve a sense of wonder that fuels creativity. There is also something profoundly valuable in encountering science without inherited familiarity. For someone who has grown up around academia, many aspects of scientific life may feel normalized. For a first-generation scientist, however, the academic world is discovered step by step. That process of discovery can preserve a sense of wonder and intellectual excitement that fuels long-term curiosity. What may initially appear as a lack of background can, in practice, become a source of renewed perspective. Finally, the experience of having limited institutional or economic capital can cultivate resourcefulness. The ability to produce meaningful and innovative research with constrained means reflects creativity and efficiency. Doing more with less is not merely a necessity; it can become a defining strength. In this way, first-generation chemists contribute not only through their scientific outputs but also through the distinctive perspectives, motivations, and forms of resilience they bring to the scientific community.

Mamta: As a first-generation chemist, I often felt mentally trained to constantly scan for the next opportunity and to secure it as early as possible, driven by a persistent sense of being behind my peers who seemed to move through academia with more ease and confidence. In that chase, it became easy to overlook my own progress, to see only the gaps and not the ground I had already covered. An important enabling factor that shifted this mindset for me was identity support that emerged through outreach. During National Chemistry Week, our Chemistry Graduate Association at the University of Rochester visited classrooms in the Rochester City School District, where the students consistently called us "scientists" and ended our scientific demonstrations by saying they wanted to be scientists just like us. Hearing myself described that way and seeing their excitement prompted a level of introspection I hadn't allowed before; for the first time, I clearly saw the distance between my humble beginnings and where I now stood. That experience made me deeply aware of how powerful community building through outreach and mentoring can be in not only quieting the impostor feelings many first-gen chemists carry, but also in enabling spaces where younger students can begin to imagine themselves as scientists too.

Douglas: I can't speak for other first-generation chemists, but I can share some general things I've learned from my own experiences that would be applicable for other first-generation chemists. Many people have to create their own path and learn how to design processes, improve workflows, and create a structured environment. I've learned to easily adapt because I was always navigating unfamiliar environments, which required me to adjust quickly, take in new information, and solve problems in different ways. This goes beyond just learning the theory for a subject. It's about the process of learning and doing science. Finally, many first-generation scientists are naturally good at other areas outside of science. My family comes from a blue-collar background and I have many skills that reflect that, including repair of scientific equipment.

Roshan: First-generation chemists often demonstrate excellence that extends beyond conventional academic measures. Because their pathways into science are frequently self-directed, excellence is often expressed through persistence,

adaptability, and the ability to navigate unfamiliar systems while maintaining scientific productivity. In my own experience, excellence has meant learning to operate across multiple dimensions of the chemical sciences, from fundamental research to applied innovation and technology translation. Working in nanomedicine and biomaterials has required not only scientific rigor but also the ability to connect chemical research with real-world healthcare challenges. Developing technologies intended for practical use demanded interdisciplinary collaboration, problem-solving under resource constraints, and long-term commitment, all of which represent important but often less visible forms of scientific achievement. First-generation chemists also often demonstrate excellence through independence and initiative. Without established academic networks, many must actively seek collaborations, funding opportunities, and mentorship. This process often cultivates strong self-motivation and a willingness to explore unconventional research directions. Another important form of excellence is the commitment to societal relevance. Many first-generation scientists are motivated to address real-world problems and improve access to scientific and technological advances. This perspective often leads to research that emphasizes affordability, usability, and practical impact alongside scientific innovation. Recognising these diverse forms of excellence would broaden the understanding of achievement in the chemical sciences and better reflect the contributions of first-generation chemists to both scientific advancement and societal benefit.

Anupam: First-generation chemists frequently demonstrate excellence in ways that conventional metrics do not capture. These include the ability to communicate scientific concepts across linguistic and disciplinary boundaries, to design and sustain community initiatives with limited institutional backing, and to mentor others through pathways they themselves navigated without guidance. In practice, this manifests in the founding of student organizations, the design of outreach programs in underserved communities, the organization of peer support networks during periods of institutional disruption, and the development of mentorship infrastructure that produces measurable outcomes, such as co-authored publications from undergraduate mentees or improved graduate student retention. These contributions represent genuine scientific and institutional value. They are also generative. For example, the scientists who build inclusive environments tend to produce research groups that are more collaborative, more creative, and more representative of the broader society that scientific research is meant to serve.

Shivani: My grandparents immigrated to the US from India in the 1970s with their children through the Immigration and Nationality Act of 1965, which granted them to ability to come regardless of a good education or profession. Growing up with a father who sometimes worked 24-hour shifts and a mother who came home from her job just to back to work to help my dad at the family convenience store, I learned a lot about perseverance and resilience from them, the most hard-working people I have ever known. This often drives me as a first-generation chemist, because both my grandparents and my parents lived a fierce life full of risk-taking and hard work to build a life for me. As such, without having had mentors within chemistry or an idea of what was possible as a chemist, I had to seek out opportunities and build relationships that otherwise would not occur. In my opinion, I believe that this is where a unique experience of excellence comes from. When you do not have opportunities presented to you or, in many first-generation chemists' experiences, non-inclusive spaces do not allow you to thrive, you still manage to seek out space for yourself when it feels as though there is no room for you. Additionally, first-generation students demonstrate excellence by bringing diverse opinions and approaches to the scientific space that fosters innovation. In my experience, they also tend to create space for other marginalized groups as they make progress in their own careers. To me, that is true excellence, because in addition to the progressive science they do, they value supporting underprivileged scientists on their own journeys.

Christian: The unique backgrounds of first-generation chemists are bringing a notable change in science. As I mentioned before, there are many voices in chemistry, and first-generation chemists are one of them. We are reshaping inclusivity in chemistry, making it tangible. Our unique stories and perspectives resonate with people with similar backgrounds and are helping bring very talented people into science. We, as first-generation chemists, are making great discoveries while mentoring other first-generation students to achieve even greater success than we have.

Brittany: Being in the online/science communicator space, I've noticed that many of the science communicators I see are also first-generation chemists who are using their platform to talk about their research, discuss topics related to the "hidden curriculum," or share about their personal/vulnerable experiences, which other people may relate to or find community in. Many first-generation chemists give back to their departmental community, such as participating as a mentor, either officially through formalized department programs such as Catalyst (https://pubs-acs-org.ezproxy.library.wisc.edu/doi/10.1021/acs.jchemed.1c00528), in affinity groups/organizations, or informally through social support (which takes up time and energy, but is not always recognized). Again, these are not commonly considered "excellence," in a traditional academic sense, but it takes a lot of energy and effort to cultivate community while also managing your own research, teaching, and job searching responsibilities. Additionally, I've noticed that many first-generation chemists now are also more open to incorporating outreach and other talents into science, or investigating research causes that are personally meaningful to them. The wide range of perspectives expands the type of thinking within the field.

Zhiling: Resourcefulness and persistence built from a nontraditional start. Interdisciplinary innovation: using LLMs for text/data mining and prediction to improve MOF crystallization quality. Mission-driven chemistry: contributing to field-tested water harvesting under extreme conditions (Death Valley test) to push scalable solutions.

4. How do you think insights from first-generation scientists may inform structural change?

David: Insights from first-generation scientists highlight the importance of transparency in academic structures. Many of the challenges encountered are not the result of lack of ability, but of lack of explicit guidance. Structural change does not necessarily require lowering standards; it requires clarifying pathways. One key improvement would be to make the academic trajectory more transparent from the very beginning of undergraduate studies. Students should be explicitly introduced to the full structure of a scientific career, from research training and doctoral studies to postdoctoral positions and professorship. Understanding the stages, expectations, and evaluation criteria early on can reduce uncertainty and empower informed decision-making. Another essential step is to clarify how scientific evaluation truly works. The role of publications, peer review, funding applications, impact metrics, and institutional expectations should not remain implicit knowledge acquired informally. Making these mechanisms explicit can democratize access to academic advancement. Mentorship structures can also be strengthened. Rather than assuming that students will intuitively understand how to navigate academia, institutions can provide structured guidance, practical workshops, and open conversations about career paths, stability, and alternative trajectories. Ultimately, first-generation scientists reveal that excellence can flourish when curiosity and resilience are supported by clarity. By making the system more transparent and intentionally accessible, institutions can ensure that talent is not limited by inherited familiarity with academia.

Mamta: Just as many lab websites already collect links for journals, data repositories, and safety protocols, research groups and PIs can also curate a small, visible section specifically for first-generation students. Collecting these resources in one place on a group website quietly signals that first gen trajectories are anticipated, valued, and supported, rather than treated as invisible or exceptional.

Douglas: My experience as a first-generation scientist shows that there are several clear opportunities for structural change. First, institutions, whether academic, government, or private, should make knowledge explicit by replacing informal gatekeep with transparent expectations, accessible documentation, and mentorship that does not assume prior knowledge about the norms. Evaluation practices can also be expanded to include process-building, mentorship, community engagement, and leadership that encourages collaboration. These are all important ways to be excellent in science. Second, it is also important to recognize and normalize diverse academic paths as these nonlinear paths into research often bring valuable perspectives. Lastly, while people will always need to deal with the academic system, we need to change the culture so that dealing with it is supported, not done alone. Departments and research groups should share the responsibility of training and mentoring students. They should make sure that knowledge is passed on intentionally, not through informal networks. The more people who participate in this work, the clearer, fairer, and more accessible the system will be for everyone, not just first-generation scientist.

Roshan: Insights from first-generation scientists highlight aspects of the academic system that are often invisible to those who have grown up within established scientific environments. Because first-generation researchers must frequently navigate academic structures without prior guidance, they can identify gaps in mentorship, access to information, and career development that might otherwise go unnoticed. One important lesson is the need for more structured and transparent guidance at early career stages. Clear information about research pathways, funding opportunities, publication strategies, and career options can significantly reduce the uncertainty faced by first-generation scientists. Formal mentorship programs that extend beyond research supervision can also help bridge knowledge gaps and provide broader professional support. Another area for structural change is the way excellence is evaluated. Expanding evaluation frameworks to include innovation, translational impact, interdisciplinary research, and societal relevance would better capture the diverse contributions of researchers from different backgrounds. First-generation scientists often bring perspectives that emphasise practical problem-solving and accessibility, which can help align chemical research more closely with societal needs. First-generation scientists also highlight the importance of inclusive research environments where individuals feel supported regardless of their background. Encouraging collaboration, reducing barriers to participation in conferences and international research opportunities, and improving access to resources can help ensure that talent is not limited by structural disadvantages. By incorporating the experiences and perspectives of first-generation scientists into institutional policies, the chemical sciences can move toward a more inclusive and dynamic research culture. Structural changes informed by these insights would not only support first-generation researchers but also strengthen the scientific enterprise by enabling a broader range of ideas, approaches, and innovations to emerge.

Anupam: First-generation scientists have direct, experiential knowledge of where institutional systems fall short and, in many cases, have developed practical interventions in response. This knowledge is an underutilized resource for structural reform. Several evidence-informed recommendations follow from this perspective. Mentorship should be formalized, funded, and assessed for outcomes rather than treated as informal goodwill; programs that pair first-generation students

with research mentors and provide structured support have demonstrated measurable impact on retention and publication outcomes. Evaluation criteria for hiring, promotion, and fellowships should explicitly recognize community-building, outreach, and mentorship as scholarly contributions rather than categorizing them as service that is acknowledged but not rewarded. Institutions should invest in language-accessible onboarding and culturally responsive programming for international and first-generation students, recognizing that these investments reduce attrition and strengthen research communities. Finally, policy development benefits from incorporating the perspectives of first-generation scientists directly through representation in governance structures, advisory committees, and curriculum design. The central argument is straightforward. The experiences of first-generation chemists are not simply personal stories, but they are diagnostic data about systemic gaps, and the field will be stronger for treating them as such.

Shivani: First-hand experiences from first-generation scientists can allow for systematic change of specific exclusive policies and practices that create non-inclusive spaces. At times, it is difficult to discern the root causes of issues in a setting where you have not experienced the issues firsthand. Therefore, listening to the experiences of these scientists and, most importantly, welcoming constructive criticism could allow us to cultivate a culture that is more inclusive of all individuals, not just first-generation scientists. Overall, these practices will not only benefit the first-generations scientists themselves, but also the community as a whole. We can recognize the benefits that first-generation chemists bring to the chemical sciences and the barriers that they encounter to achieve this "excellence". I believe it is in everyone's best interest to create a welcoming space for individuals from all walks of life to have the greatest collaborative environment to advance science.

Christian: Early exposure to science during childhood is important for encouraging more young people to pursue careers in science. When I was a kid, access to information was limited, especially in a modest-income home. In the early 90's, an internet service was more of a luxury than a basic necessity, as it is today. If it were not for my grandfather's encyclopedia, I may not even be a scientist today. As a first-generation chemist, I lacked early exposure to science. Fortunately, I had the fortune to meet outstanding mentors who helped me overcome that and become what I am. I bet thousands of kids haven't had that opportunity. This is where our insights as first-generation scientists can make a difference. We have experienced the same barriers. We come from similar backgrounds and understand the society around those young people. We understand the different directions that can be taken to unlock their full potential. Giving first-generation scientists a voice as ambassadors to bring more young people into scientific fields is especially important nowadays, when social media has amplified noise and misinformation.

Brittany: One example of this is https://www.cientificolatino.com/ - a program that is run by first-gen scientists, helping future scientists. I am not a part of this program right now, but I have used their resources before, when I was in the process of applying. Now that there many more first-generation scientists, there are more resources about getting into graduate school and support networks for people to lean on when they are in graduate school.

Vishal: Insights from first-generation scientists highlight several opportunities for improving the structure of academic chemistry. One important step is greater transparency. Many aspects of academic progression, grant writing strategies, networking practices, or career navigation, are often transmitted informally. Making these processes more transparent can reduce barriers for individuals who lack access to informal academic networks. Another key change is valuing mentorship and community contributions as part of academic excellence. Recognizing these contributions in hiring, promotion, and funding decisions would encourage a broader culture of support within the field. Finally, institutions should continue expanding structured mentorship programs, funding support for trainees, and inclusive recruitment practices. These initiatives do not lower standards; rather, they help ensure that talented scientists from diverse backgrounds have equitable opportunities to succeed. Ultimately, broadening how we define and support excellence will strengthen the chemical sciences by enabling a wider range of scientists to contribute their ideas, perspectives, and creativity.

Zhiling: Make mentorship and career-navigation knowledge explicit (not informal). Broaden "excellence" criteria to value interdisciplinary tool-building, field translation, and education/outreach. Early access matters: I helped bring MOF crystallization into high school classrooms and used AI as a "digital mentor" in STEM education to make advanced chemistry more accessible.

General responses covering points 1–4.

Adam: It takes a village to raise a scientist.
As a first-generation university student from a prohibitively low-income household, I've come to deeply value the role that principles – such as responsibility, innovation, respect and self-confidence – play in one's personal and scientific development. In fact, I sometimes refer to myself as a "turbo first-generation student", as no one in my immediate family has graduated high school, let alone earned a PhD. Today, I'm a postdoctoral fellow at Stanford preparing to launch an academic career and looking back on the path that brought me here, two *guiding light principles* stand out. First, teams that reflect the globe generate the kinds of ideas necessary to approach global challenges. And second, diversity in viewpoints – sociopolitical (as is often the case of the first-generation student) or otherwise – is essential in academia as it is what

forms the foundation of belonging, access, responsibility and mentorship, four ideals that I try to emphasize in my everyday career that trace back to my beginnings as a first-generation student.

The value of belonging: Who am I?

The foremost barrier I've experienced throughout my journey comes across in one simple question: *Do I really belong here?* For many, heading to university means leaving "the life before" behind – of course, this is part of growing up, but for me it meant something deeper. Choosing to attend university ranks among the most incredible decisions I have made in my life, but even just the act of that choice was a watershed moment in my development. Not a single one of my closest friends from high school pursued a postsecondary education – these were the people I had grown up with, the ones who had stood by my side for the most incredible as well as the most traumatic coming-of-age-type events in my life. Sure – I've kept in contact with a select few of these folks, but there was no question that the life I had lived up until I stepped into a university hallway was incompatible with all that would follow. Birds of a feather, too, as when I began my first semester of university, I found myself in a world that I had never been prepared for, surrounded by peers who I didn't relate to in an environment that was foreign at best and perceived as hostile at worst. Learning chemistry was a challenge – learning how to exist in a university setting was another entirely. *I didn't know what I didn't know*, and I felt like a complete outsider. Even the very concept of being a "first-generation student" wasn't understood until a few years down the line.

As time went on and I grew into the post-secondary environment, things changed and – as first-generation students tend to do – I learned to adapt. Even now, thirteen years later, I find myself asking the same question – *do I belong here*? Looking back on those early days, which were mimicked by my early days in graduate school as well as those as a postdoctoral researcher, it has been a real series of 'stepping into the world of colour TV' type moments.

Considering excellence in the sciences: Accessibility is not just a buzzword

One form of excellence that first-generation students demonstrate is a true commitment to the goal of making science accessible. Related to the above comments on belonging, something I have consistently had to navigate during my life is explaining what I do – synthetic, mechanistic chemistry sprinkled with hints of chemical biology – to family members and friends who have no *educational* background, let alone a *scientific* one. While challenging, this became one of my most incredible strengths as it has forced me to learn how to translate scientific concepts into language that people beyond the walls of a university can understand. And wow – is this ever an important skill to have today as the *age of misinformation* rears its head in various aspects of our discipline. Many of the solutions to the grandest problems of our time depend on public understanding of science, and because first-generation scientists often remain closely connected to communities outside of academia, we develop a natural ability to communicate across the divide. We understand what life is like on the other side of the coin, so to speak. For this reason, there is one word in academic presentations that I absolutely cannot stand: "obviously". Because nothing we do in science is obvious. Defining "excellence in academia" is something that I have always struggled with. In the most traditional sense, it can be measured by grades, publications, awards and citations – which, of course, matter. But my first-generation experiences have led me to view success under a different light – for instance, from my family's perspective I am not introduced as someone with X amount of publications and Y awards. I am "Adam with the PhD". And this perspective matters. If I were to reconsider excellence in academia, I would put more emphasis on the baseline. *First-generation students have to travel through a desert just to reach the starting line*. And even once that starting line is reached, we often find that we've arrived without a map for what comes next. Publishing ten thousand *Science* or *Nature* papers – in my mind – pales in comparison to the excellence associated with inspiring others to take that same path through the desert, especially those who might not have imagined it possible. I would redefine excellence in the chemical sciences as impact on the world – *impact on people* – rather than impact factor in journals. Using your career as a platform to inspire interest in science is every bit as "excellent" as becoming a scientist yourself.

A core tenet of my teaching philosophy, the "*sports coach approach*", reflects this view and originates from my experiences as a first-generation student. I try to teach as a coach would: encouraging students to focus on reaching their full potential, emphasizing the value of teamwork and reassuring them that the worst-feeling failures lead to the most gratifying success. *The only person a student should worry about competing with is the person that they were the day before*. These are the words that I wish someone had told me when I first arrived at university thirteen years ago. Unfortunately, these are also the words that are often forgotten in the quest for academic excellence, as traditionally defined.

Responsibility and Mentorship

For first-generation students, mentors often become much more than academic supervisors. Because as first-generation students, we often lack individuals that can *show us the ropes*, so to speak. Perhaps the most printed sentence in academic theses begins with something along the lines of "I'd like to thank my advisor", but for first-generation students, that gratitude runs deeper. If it were not for a handful of extraordinary mentors – at OntarioTech, the University of Ottawa, and Stanford – then I would not be where I am today. Mentors become role models for a world we knew nothing about – like the spaceman who takes your hand during your first steps on some far away planet. This is the beating heart behind my desire to continue my journey in academia – to help guide students who might otherwise feel lost within the academic system. As a first-generation student, I have learned firsthand the *responsibility* of becoming a mentor. And responsibility manifests in another way for many first-generation students: *responsibility to family.* The pursuit of higher education is not just a way to spend our twenties. For many of us, it is not just a pathway to stability, but *the* pathway toward stability – not only for ourselves but also for those around us. Beyond the financial stresses often associated with being first-generation,

there can be an underlying sense of responsibility for first-generation students wherein they become a kind of lifeboat for the rest of the family – the one tasked with parental care in their later years, or the one responsible for cleaning up the messes of others. This creates a lot of pressure to succeed, but it also instills a deep appreciation for education itself. I have always been very much aware that education is the single most powerful tool that has allowed me to change the trajectory of my life, and I believe that first-generation students share a particularly close-to-heart understanding that education is a privilege – something that should never be taken for granted. In the acknowledgements of my PhD thesis, I write that *earning a PhD felt like an act that flies in the face of predetermination*. One small decision in another direction, and none of this would have happened. This perspective of first-generation scientists shapes how challenges are approached. Having *looked at clouds from both sides now*, to paraphrase fellow Canadian Joni Mitchell, we understand that when an experiment fails or product comes off of a column as impure, life will go on. Resilience, self-confidence, gratitude and commitment to mentorship – these are just some of the forms of excellence exuded by first-generation scientists.

Navigating the invisible curriculum and how insights from first-generation scientists can inform structural change

Amongst the most significant challenges that first-generation scientists face is navigating the invisible curriculum of academia. A remarkable number of skills that are essential for success – grant writing, professional communication, even understanding what careers in science look like – are rarely taught explicitly.

One story I like to mention recalls when I first arrived at university and realized that there was an entire field called *engineering*. Before that moment, the word "engineer" meant – to me – someone who drove trains. While humorous, this story illustrates something incredibly important: first-generation students often arrive at university with curiosity and motivation but without the background knowledge that others have absorbed informally. The danger of this, of course, is that these gaps in knowledge can be misinterpreted as ignorance, which discourages students from asking the questions that they need answered the most. Stemming from the oft-fact that first-generation scientists often have to get where they are by themselves, this line of thought can result in fiercely independent students. Which is not always a bad thing. But, as a mentor once told me: it doesn't matter if you build the perfect, most incredible bridge – it if it leads in the wrong direction, you're destined to be swimming.

If we want to support first-generation scientists effectively, *transparency has to be a goal.*

One thing I would love to see is a public repository of scientists willing to simply have conversations with first-generation students. Whether it be in class or in research, first-generation students often do not require help with the science – what we lack is access to the hidden norms, expectations and procedures that enable others to navigate academic systems more efficiently. Speaking for myself, I sure could benefit from just having someone to reach out to that I could ask the questions that I feel are often assumed knowledge, free of judgement.

First-generation students do not lack ability, and they do not lack motivation. What we lack is access and a community that understands what it really means to start from a blank slate. I can imagine an online seminar or a short course for incoming students designed to illustrate transparently what we should implicitly know coming into university. *Outreach programs geared towards first-generation students are important, but equally important are in-reach programs designed to ensure that first-generation students have the structures and support needed to thrive.* Much of academia is hidden in bureaucracy or within words and behaviours that are simply not accessible, or familiar, to first-generation students. I would love to be part of a world where "first-generation mentors" are as common in the halls of post-secondary schools as research mentors. Because when someone is dropped into a body of water, sometimes all it takes is a hand from a boat to grab on to.

Without knowing whether one has fallen into a puddle or an ocean, it is all too easy to sink.

Nitesh: I vividly remember the moment I declared against all expectations that I wanted to become a doctor. It was the first time anyone in my family had voiced such an ambition. Most people laughed at my ambition: friends, relatives, neighbours, everyone except my father. I did not become a medical doctor in the conventional sense; instead, I became a Doctor of Philosophy, earning a PhD in interdisciplinary medicinal chemistry and neuroscience from the College of Pharmacy, the University of Manitoba, Canada. Along the way, I lost my father but never his belief in me. Those who laughed were not wrong. In my family, no one had the privilege to complete high school. There was no legacy of education, no financial security, no academic environment, no mentors, or, as we say in India, no Guru to illuminate the path. Yet my father understood something deeply. He embodied resilience (R), hard work (H), perseverance (P), commitment (C), passion (P), and dedication (D). He believed that with these principal values, what I now think of as continuous "RHPCPD," it is possible to break generational cycles and inspire future generations.

He once told me, repeatedly:

"Nitesh, I can buy you books and pay your fees, but I cannot mentor you regarding science education. I do not know science. You must do this yourself. Otherwise, you can join me in the cloth shop."

Those words carried both support and responsibility. With his continuous encouragement, I became the first person in my family to pass grade 10 in India. I still remember that day vividly: many of my friends in the neighbourhood could not pass grade 10, while I passed with first division, scoring over 90/100 in science. I can still picture my father's pride. That moment gave me the confidence to pursue science in grade 12, taking Biology, Chemistry, and Physics, with the dream of becoming a physician. Still a big decision for my family, many friends and family members told me to pursue different streams, like accountancy. Many told me to sell clothes in my father's shop. The real struggle began afterward. In 1998, after passing grade 12 with first division, I faced the national-level medical entrance examinations. My father supported

me in attending coaching classes in a distant metropolitan city. I promised him I would not let him down, even as he worked day and night to pay my fees. I lived on one meal for several days, balancing rent, coaching fees, and transportation. I fell seriously ill several times with severe stomach pain. I failed the medical entrance exam not once, but year after year. I spent six years trying. During this time, I faced shame, isolation, weight gain, and deep emotional distress. I stopped attending social gatherings. Friends and family told me I was wasting my father's money and effort. Sometimes I thought about the value of my life. Yet one person never lost faith: my father. Other than the medical stream, I qualified for top schools for pharmacy, agriculture, and other streams. I dropped other streams because there was no one to guide me for these streams, which I realized later in life. And I continued my aspirations to become a doctor.

Eventually, under immense pressure, after dropping for six years, I enrolled in a Bachelor of Pharmacy program at SHUATS (Allahabad), India never my original dream, but a turning point. There, I discovered I was not alone; many students had also spent years chasing medical school. Seniors encouraged me, tested my knowledge, and introduced me to NIPER, the National Institute of Pharmaceutical Education and Research, a premier centre of excellence in India. For the first time, I saw another path to making an impact in science and medicine. I studied relentlessly while earning my pharmacy degree, studying during vacations and enduring power outages by candlelight, alone when others went home. I always remember my father's sweat, so I believed that the more I sweat in summers under melting candles, the more successful I would become. I always had in my mind "Nitesh, this is the only chance, and I cannot afford to lose it." In 2010, I qualified for both GPAT and NIPER, two highly competitive national examinations. When I told my father that I got qualified for NIPER, his voice carried pride and relief: "Nitesh, you did it."
Our community, once filled with doubt about me, came together to organize a heartfelt celebration in honor of my father. That moment was purely his, a testament to the impact he had. My father was on cloud nine, overwhelmed with pride and joy that day.

Reflecting now, I see the systemic barriers clearly: financial strain, lack of mentorship, lack of trust, isolation, societal pressure, and the harmful framing of education as a status symbol. In many middle- and lower-income communities, educational success becomes a social currency, leaving those without privilege feeling inferior, discouraged, and excluded. True excellence, I believe, must be redefined. Education should never be a marker of social superiority; it is both a privilege and a fundamental human right. Those with educational access must take responsibility to uplift underrepresented communities, mentor intentionally, and create equitable environments where success is collective, not competitive. Over time, I came to realize that communities should not elevate certain professions into symbols of social status, as this often fosters comparison without compassion and reinforces inequities rather than shared values. Early in my journey, I, too, saw respect narrowly defined, reserved mainly for physicians and a few traditionally esteemed roles.

With experience and reflection, my perspective evolved. I learned that meaningful change in human lives is not limited to any single title or profession. Every vocation carries dignity, responsibility, and the power to serve others in profound ways. Whether visible or behind the scenes, each profession contributes essential values that sustain society. True respect, I now believe, lies not in hierarchy, but in purpose, integrity, and the impact one chooses to create. Today, I never forget where I began. I am a first-generation scientist from Jharia, a small coalfield in India. I am now a Canadian citizen, a medicinal chemist, a postdoctoral fellow at the University of Manitoba, a mentor, an inventor, a co-founder, and a father of two daughters. In December 2025, I completed my PhD under Dr. Geoffrey K. Tranmer, and I continue my work driven by a mission to accelerate therapeutic discovery, elevate patient voices, and reshape the scientific landscape, particularly for those living with incurable diseases such as ALS. My journey from grass to grace has taught me that excellence is not about pedigree or privilege, but about perseverance, responsibility, and using one's success to create hope for others.

I believe education is both a privilege and a profound responsibility, an opportunity to bring goodness and positive change into human lives. Around the world, many are denied this chance, not because of a lack of potential, but a lack of access. As a first-generation scientist, I recognize how rare and transformative this privilege has been for me. Education did not simply open doors for my own growth; it gave me the ability to change the trajectory of my family's future and to create possibilities for generations to come. I carry this journey with gratitude, humility, and a deep commitment to use my learning in the service of others.

References

S1 Naddaf, M. Is academic research becoming too competitive? *Nature* **2025**, *646* (8087), 1036–1037.
S2 Universities are – and must continue to be – a force for good. *Nature* **2025**, *645*, 821.
S3 Simms, C. Pressure to Publish Is Rising as Research Time Shrinks, Finds Survey of Scientists. *Nature* **2025**, *647*, 565–566.
